# Common loss of far-red light photoacclimation in cyanobacteria from hot and cold deserts: a case study in the *Chroococcidiopsidales*

**DOI:** 10.1038/s43705-023-00319-4

**Published:** 2023-10-19

**Authors:** Laura A. Antonaru, Vera M. Selinger, Patrick Jung, Giorgia Di Stefano, Nicholas D. Sanderson, Leanne Barker, Daniel J. Wilson, Burkhard Büdel, Daniel P. Canniffe, Daniela Billi, Dennis J. Nürnberg

**Affiliations:** 1 Institute for Experimental Physics, Freie Universität Berlin, Berlin, Germany; 2 Department of Life Sciences, Imperial College London, London, UK; 3 Dahlem Centre of Plant Sciences, Freie Universität Berlin, Berlin, Germany; 4 Department of Integrative Biotechnology, University of Applied Sciences Kaiserslautern, Pirmasens, Germany; 5 Department of Biology, University of Rome Tor Vergata, Rome, Italy; 6 PhD Program in Cellular and Molecular Biology, Department of Biology, University of Rome Tor Vergata, Rome, Italy; 7 Nuffield Department of Medicine, University of Oxford, Oxford, UK; 8 NIHR Oxford Biomedical Research Centre, Oxford, UK; 9 Big Data Institute, Nuffield Department of Population Health, University of Oxford, Oxford, UK; 10 Department of Biology, University of Kaiserslautern, Kaiserslautern, Germany; 11 Institute of Systems, Molecular and Integrative Biology, University of Liverpool, Liverpool, UK

**Keywords:** Environmental microbiology, Microbial ecology

## Abstract

Deserts represent an extreme challenge for photosynthetic life. Despite their aridity, they are often inhabited by diverse microscopic communities of cyanobacteria. These organisms are commonly found in lithic habitats, where they are partially sheltered from extremes of temperature and UV radiation. However, living under the rock surface imposes additional constraints, such as limited light availability, and enrichment of longer wavelengths than are typically usable for oxygenic photosynthesis. Some cyanobacteria from the genus *Chroococcidiopsis* can use this light to photosynthesize, in a process known as far-red light photoacclimation, or FaRLiP. This genus has commonly been reported from both hot and cold deserts. However, not all *Chroococcidiopsis* strains carry FaRLiP genes, thus motivating our study into the interplay between FaRLiP and extreme lithic environments. The abundance of sequence data and strains provided the necessary material for an in-depth phylogenetic study, involving spectroscopy, microscopy, and determination of pigment composition, as well as gene and genome analyses. Pigment analyses revealed the presence of red-shifted chlorophylls *d* and *f* in all FaRLiP strains tested. In addition, eight genus-level taxa were defined within the encompassing *Chroococcidiopsidales*, clarifying the phylogeny of this long-standing polyphyletic order. FaRLiP is near universally present in a generalist genus identified in a wide variety of environments, *Chroococcidiopsis sensu stricto*, while it is rare or absent in closely related, extremophile taxa, including those preferentially inhabiting deserts. This likely reflects the evolutionary process of gene loss in specialist lineages.

## Introduction

Environment shapes evolution, in both the macro- and microcosmos. Among bacteria, the oxygenic photosynthetic cyanobacteria have diversified to occupy numerous niches by synthesizing specific pigments, forming biofilms, or fixing atmospheric nitrogen [1, 2]. Early colonizing cyanobacteria are often the most significant carbon inputs in extreme, nutrient-poor environments, including deserts [3, 4]. Defined in this work as (hyper-)arid environments (Aridity Index <0.2, or Potential Evapotranspiration <400 mm for cold deserts) [5], they encompass places as varied as the Atacama Desert and University Valley in Antarctica, and are often subject to additional stresses, such as high or low temperatures, large daily temperature variations, ice crystal nucleation, and intense solar radiation [4]. For this reason, many desert cyanobacteria colonize the subsurface or the interior of rocks [4, 6, 7].

A microorganism commonly inhabiting deserts is the cyanobacterium *Chroococcidiopsis*. This genus is characterized by non-motile solitary spherical cells forming non-polarized agglomerations, and the ability to reproduce by non-motile baeocytes [8, 9]. It has been isolated from cold and hot deserts, hot springs, and spacecraft assembly clean rooms [7, 10–16], with some isolates even surviving space [17, 18]. Previous studies have highlighted extreme temperature tolerance as a lineage-specific trait, with different lineages characteristic of hot or cold deserts, respectively [15, 19]. Nevertheless, strains have also been found in seemingly moderate environments [12, 20, 21]. This environmental diversity, combined with the large number of strains available from culture collections, make *Chroococcidiopsis* an ideal test subject for the study of adaptations to extreme environments.

It has become clear that at least some members of the *Chroococcidiopsis* genus can also thrive in the absence of visible light, for instance in soil, biofilms, or within sedimentary rocks, by using far-red light / near-infrared radiation (~700–750 nm) [16, 22–26]. In order to do so, they undergo a process called far-red light photoacclimation (FaRLiP). This involves the extensive remodeling of the photosynthetic apparatus and the synthesis of new pigments, namely chlorophyll *f*, as well as traces (<1%) of chlorophyll *d* [23, 27, 28]. The significance of chlorophyll *d* in particular is an unsolved puzzle, as some studies locate it in the reaction center of far-red Photosystem II [23, 29], yet many strains appear to lack it altogether [21, 30–34]. In addition, FaRLiP relies on a 20-gene cluster encoding paralogues of Photosystem I, Photosystem II and phycobilisome components, as well as a phytochrome regulatory cascade [24]. It is present in a small but diverse range of cyanobacteria, and is mainly associated with shading by other photosynthetic organisms [22, 24]. However, certain types of rock such as quartz may create similar microenvironments through the preferential transmission of longer wavelengths [6, 16]. As desert *Chroococcidiopsis* strains have been frequently isolated from lithic habitats, they might provide models for understanding not only the evolution of far-red light photoacclimation, but also its role in the environment.

Some *Chroococcidiopsis* lineages are either hot or cold desert specialists [15, 19]. Other strains from moderate environments might be generalists, based on the wide spread of this genus [12, 20]. Comparing their FaRLiP capacities could highlight selective pressures and constraints for the habitat. Nevertheless, recent research casts doubt on taxonomically assigning certain extremophilic lineages to the same genus. For example, the lineage known as “Cold Desert *Chroococcidiopsis*” has recently been typified as the separate genus *Aliterella* [35]. Still, there is strong evidence of FaRLiP being present in the last common ancestor of *Chroococcidiopsidales* (the order containing *Chroococcidiopsis*), thereby motivating our research [22, 36]. Traditionally, many related strains have been intermixed and simplified as “*Chroococcidiopsis*”. Recent research has been addressing this topic, but an overarching view that combines fine-scale 16S rRNA gene, genomic and ecological data is still missing [37–40].

The availability of cultured strains sampled from all over the world, together with the recent expansion in sequencing data, including metadata and metagenome-assembled genomes (MAGs), enabled this investigation. Therefore, this work presents a fine-grained evolutionary history of far-red light photoacclimation within the genus *Chroococcidiopsis*, and reveals a stark contrast in FaRLiP maintenance in generalist versus specialist taxa. In the process, this work also clarifies phylogenetic relationships within this genus and its associated order.

## Materials and methods

### Cyanobacterial strains and cultivation

The 43 cyanobacterial strains were obtained from the culture collections PCC, SAG, CCALA, CCMEE, and BCCM/ULC (Table S1). The last two provided the extremophilic strains. All strains were grown in BG11 [9] at room temperature, apart from Antarctic BCCM/ULC strains (10 °C). Far-red light incubators used LEDs centered at 750 nm (LED750-03AU, Roithner). Cultures were grown in parallel under white light. The photon flux ranged between 10 and 30 μmol photons m^–2^ s^–1^.

### Fluorescence microscopy and spectra

In order to investigate photosynthetic acclimation, fluorescence images and spectral scans of cyanobacterial samples were acquired with an inverted confocal laser scanning microscope (CLSM; Leica SP8) using a HC PL APO x63/1.40 oil CS2 objective. Strains were grown for more than 20 days under FRL, immobilized onto BG11 medium containing 1% agar (w/v) and excited at 488 nm. Fluorescence images were taken by detecting the emission from phycobilisomes and chlorophyll *a* at 660–700 nm and from chlorophyll *f* at 720–750 nm. CLSM lambda-scans were obtained by collecting the emission from 550 to 800 nm with 3 nm steps and 5 nm bandwidth. Images were analyzed with Leica LAS X (version 3.5.6) and FIJI [41]. Spectral analysis was performed using Jupyter Notebook (version 6) running Python 3.

### Pigment analysis

Pigments were extracted and analyzed by HPLC using an Agilent 1100 HPLC system. Samples were run on a Supelco Discovery HS C18 column (5 μm particle size, 120 Å pore size, 250 × 4.6 mm) at 1 ml·min^–1^ and 40 ˚C. Solvent A was 64:16:20 (v/v/v) methanol:acetone:H_2_O, while solvent B was 80:20 (v/v) methanol:acetone. Solvent B was held at 50% for the first 2 min, increased linearly to 100% over 10 min, and was held there for 25 min. Elution of chlorophylls *a*, *d*, and *f* was detected by monitoring absorbance at 665, 696, and 705 nm, respectively.

### DNA extraction and quantification

Genomic DNA (gDNA) was extracted using Quick-DNA Fungal/Bacterial Miniprep Kit (Zymo Research), with a longer bead-beating time (20 min). DNA concentration was measured with a NanoDrop spectrometer or a Qubit fluorometer (Thermo Fisher Scientific).

### Amplification of marker genes

The ability of strains for FaRLiP was assessed by amplifying the far-red specific marker gene *apcE2* by PCR from genomic DNA. This has been previously shown to be a simple, reliable assay in a wide variety of species [22, 42]. Two additional primer sets were used for the amplification of 16S rRNA gene and for the hypervariable intergenic sequence (ITS) between the 16S and 23 S rRNA genes. The PCR and extraction protocol used was the same as published before, with 25 ng gDNA per 25 µl reaction [22]. The amplicons obtained were sequenced (Microsynth Seqlab). For *apcE2* amplicons in particular, 5ʹ tags were used as sequencing primers. All primers are listed in the Supplementary Material (Table S2).

### Single-gene phylogenies and rooting

In addition to the amplicons obtained above, 16S rRNA and *apcE2* gene sequences were also recovered from NCBI [43, 44]. Search settings for *apcE2*: BLASTp (nr database) and tBLASTn (WGS database, “cyanobacteria”). Search settings for 16S rRNA genes: BLASTn, nr, 1000 results. Query: 16S rRNA of *Chroococcidiopsis thermalis* PCC 7203. Sequences were aligned with MAFFT using Jalview [45, 46]. Phylogenies were built with RaxML on the CIPRES webserver [47, 48]. Settings: RAxML-HPC2 on XSEDE, model GTR CAT, bootstrap iterations 100. Trees were edited with iTOL [49] and Inkscape 0.92. For the graphical abstract, BioRender was used. Networks were built with Splitstree for alternative visualization [50]. Large branches with uncertain labels (e.g. “uncultured bacterium”), or outside the scope of this study, were removed. So were 16 rRNA genes showing high similarity to *Chroococcidiopsis* sequences, and assumed to be close relatives in previous work [38], but revealed by genome phylogenies to be distantly related [36]. “*Chroococcidiopsis*” sequences belonging to different orders were also largely filtered out (Table S8). Additional divergent sequences, including partial sequences (700 nt) were recovered through BLAST searches, for a total alignment length of 1284 ± 169 bp. For the *apcE2* phylogeny, the root point was confirmed with Minimal Ancestral Deviation (MAD) [51] and previous work [22].

For the 16S rRNA gene phylogeny, rooting was done with *Pleurocapsa* sp. PCC 7327 as an outgroup. This strain is capable of FaRLiP, and branches out from just outside the group containing the *Chroococcidiopsidales* together with their closest relatives, the *Nostocales* [24, 36]. Except for the position of the *Nostocales* as an artifact in 16S rRNA gene trees, the branching pattern closely mirrored the genome tree and was therefore regarded as overall more accurate [36]. Using either *Nostocales* or *Gloeobacter* as an outgroup significantly changed this branching pattern, and was therefore considered less accurate. Previous work also shows this branching instability [15, 35, 37, 38, 52].

### Sequence comparison of 16S rRNA genes

Simple sequence similarity can be a useful phylogenetic proxy. The 16S rRNA genes were aligned with MUSCLE in a ClustalW format [53]. The output was submitted to Clustal Omega in order to generate a percentage identity matrix (PIM) [54]. The matrix was parsed and statistics calculated with Python 3.7 using PyCharm (modules: pandas, statistics) [55].

### RNA structure prediction

D1-D1’ and Box B loop structures from the ITS were predicted by running SPOT-RNA on a Linux server [56]. This included non-canonical base pairs/pseudoknots stabilized by tertiary interactions. Results were cross-checked with Mfold and RNA-fold [57, 58].

### Genome data sourcing: sequencing and assembly

Based on their phylogenetic position, *Chroococcidiopsis* sp. SAG 39.79, SAG 2023, SAG 2025, and *Chroococcopsis gigantea* SAG 12.99 were sequenced using Illumina and Oxford Nanopore Technologies (Table S3). Samples were sequenced according to vendor protocols on a MiSeq and MinION (MinKNOW version 1.10.23), the former using Nextera XT v2 and the latter a R9.4.1 flowcell with base-calling via Albacore (version 2.1.7). In addition to these samples, a metagenomic dataset from the Atacama Desert (NCBI codes SRR2394720 and SRR2396013), associated with ignimbrite rock, was reassembled. Its sourcing is described in the following section.

Trimming and quality control was performed with BBDuk (settings: qtrim=r trimq=10 minlen=30 ktrim=r k = 23 mink=11 hdist=1 tpe tbo) [59]. Assembly was executed for each individual sample with MEGAHIT (settings: meta-sensitive, paired) [60] for the metagenome, and the hybrid assembler Unicycler for the cultured strains [61]. Samples were co-binned with vamb [62]. CheckM, Prokka, and the BBMap statswrapper module were used to estimate bin quality after refinement with anvi’o [59, 63–65]. The SAG 39.79 assembly was further improved with LongStitch (settings: tigmint-ntLink-arks) [66].

### Genome-level analyses

Besides the locally sequenced samples, genomes were recovered by genus name, as well as by BLAST searches [43]. For clades with little to no representation, four additional metagenome bins were obtained: “ignimbrite12”, “ignimbrite01”, “Atacama+Negev” and “mojave”. The first two are associated with samples from the Atacama Desert (Fig. S3) [67]. Their raw data was shown to contain *apcE2* fragments corresponding to *Chroococcidiopsidales* in a previous study using SearchSRA for data mining [22, 68]. Bin “Atacama+Negev” was identified from the literature as *Chroococcidiopsis* [16], and confirmed with a genome tree. It is representative for three near-identical FaRLiP endolithic bins from the Atacama and Negev deserts (IMG/MER bin used 3300037877_1; similar bins 3300039401_1 and 3300039404_1) [16]. Bin ‘mojave’ was originally sampled from the Mojave desert, and recovered with IMG/MER’s 16S rRNA-based BLAST search (original IMG/MER bin ID 3300034134_4; permission granted by Kirsten Fisher from California State University, USA) [69].

The genome phylogeny was built with OrthoFinder, and included 13 strains which belong to the *Chroococcidiopsidales* [70]. Orthofinder uses proteome inputs; where not already available, these were obtained from genomic sequences with Prokka [65]. Despite the different genus name, both the 16S rRNA gene and the genome labeled *Scytonema millei* VB511283 (JTJC00000000.3) clustered with *Chroococcidiopsis* strains.

To identify clade-specific genes, the genomes and metagenome bins were submitted to OrthoVenn2 [71]. Datasets chosen as representatives for their respective clades: *Chroococcidiopsis thermalis* PCC 7203, *Gloeocapsa* sp. PCC 7428, *Aliterella atlantica* CENA595, the original ignimbrite12 metagenome, the mislabeled *Scytonema millei* VB511283, *Chroococcidiopsis* sp. CCALA 051, Chroococcales cyanobacterium IPPAS B-1203, Mojave metagenome. As ignimbrite12 was the only representative of its clade, clade-specific genes were approximated by dataset-specific genes. Default settings. The orthologue sets recovered were submitted to BlastKOALA and KofamKOALA on the KEGG webserver [72, 73], with KEGG Mapper enabling a better understanding of genus-specific functional pathways [74].

### Environment classification

Desert environments were classified into hot and cold depending on the main reason for their aridity: evaporation or low temperatures, respectively [5]. This has been typically used in cyanobacterial work [15]. Equivalents in the Köppen–Geiger climatic scheme: Bwh for hot deserts, and ET (polar or alpine) for cold deserts [75]. For the purposes of this study, the mild Atacama Desert was considered ‘hot’. In addition, a subset of hot deserts with large seasonal variations, where average monthly temperatures might drop below 0° C, were additionally labeled “hot-and-cold” [76]. This category also included sampling sites from unspecified deserts. For 16S rRNA genes, all NCBI metadata was considered, together with citing literature when necessary / available.

## Results and discussion

### Red-shifted chlorophylls d and f are extensively present in the genus Chroococcidiopsis

A broad range of 43 samples labeled as *Chroococcidiopsis* were obtained from culture collections. These strains are representative of either non-extreme environments (19) or deserts (20 from hot deserts, 4 from cold deserts) (Table S1). The strains were tested for their ability the perform FaRLiP by using a combination of molecular biology, biochemical and biophysical methods. A first screening approach was performed by PCR for the FaRLiP marker gene *apcE2* (Table S2). This gene has always and exclusively been found co-occurring with *chlF* (*psbA4*), the gene encoding the chlorophyll *f* synthase [22]. Out of the 43 tested strains, 22 were positive for the presence of *apcE2*.

**Fig. 1:**
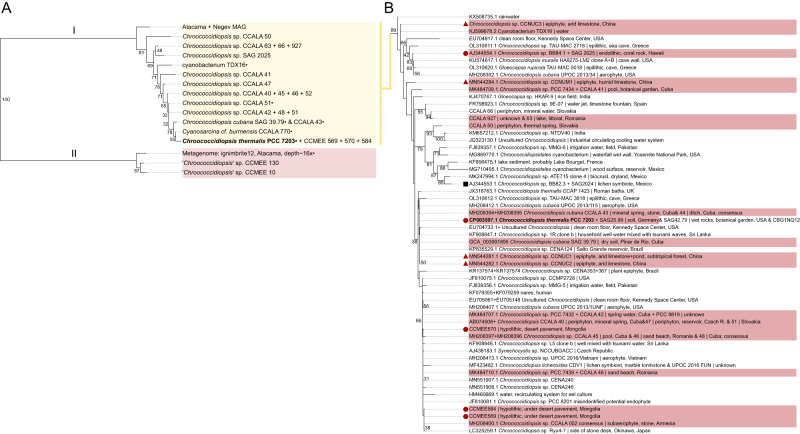
Distribution of FaRLiP in the order *Chroococcidiopsidales*. **A** Phylogeny of ApcE2 marker sequences from *Chroococcidopsis* strains, or strains previously classified as such. Bullet points mark full-length proteins. There is a clear split between two branches, I and II, with the largest one (I) associated with *Chroococcidiopsis sensu stricto*. Type species *Chroococcidiopsis thermalis* PCC 7203 is listed in bold. **B** Distribution of FaRLiP in the genus *Chroococcidiopsis sensu stricto* (I). The 16S rRNA gene phylogeny includes strains which tested positive for *apcE2* and/or strains with a known FaRLiP cluster (red). Most of them also survived under far-red light, except for a minority of non-axenic strains (CCALA 44, 47, 51, 52, 927). One strain tested negative for *apcE2* and did not survive under FRL (black square). HPLC data showed the presence of chlorophylls *d* and *f* (circles) or only chlorophyll *f*, as reported in previous studies (triangles). Trees built with RaxML. Bootstrap values < 30 not shown.

A phylogenetic tree was built with the amino acid sequences encoded by these 22 *apcE2* genes, together with homologs recovered from the NCBI. The ApcE2 phylogeny is split into two main branches (Fig. 1A: I and II). The majority of sequences fall in group I, and are associated with a narrow definition of *Chroococcidiopsis* around the type strain *Chroococcidiopsis thermalis* PCC 7203 (hereafter referred to as *Chroococcidiopsis sensu stricto*). These strains have been largely sampled from non-extreme environments (Fig. 1B). However, sequences for “*Chroococcidiopsis’* CCMEE 10, CCMEE 130, and ignimbrite12 (a metagenomics sequence from ignimbrite rock in the Atacama Desert) are clearly divergent from the others (Fig. 1A: II). These strains have been sampled from hot deserts and appear to belong to a different genus, yet to be defined.

The ApcE2 phylogeny (Fig. 1A) matches the 16S rRNA gene phylogeny (Fig. 1B), the genome tree discussed later, as well as a higher-resolution *Chroococcidiopsis* tree based on MALDI-TOF data [20]. These results are consistent with vertical descent driving the distribution of FaRLiP within this genus, as opposed to horizontal gene transfer as has previously been suggested for this phenotype [24]. As the *apcE2* gene is an indirect, though fast marker for FaRLiP [22], additional methods were used to assess the cellular response. These included monitoring the long-term survival of the strains under far-red light (as judged by pigmentation), recording fluorescence emission spectra and determining pigment composition by HPLC. Out of the 22 *apcE2*-positive strains, 18 strains were selected for further analyses as representative of phylogenetic and environmental variation. Out of these, 13 strains survived in far-red light (Table S1). They included 11 strains from group I and 2 from group II. The remaining 5 cultures (group I) showed abundant non-cyanobacterial growth, which likely outcompeted the cyanobacteria under the test conditions. FaRLiP is a slow process, taking 12-14 days for full acclimation, during which cyanobacteria do not grow [77]. None of the 8 *apcE2*-negative strains tested survived these conditions.

**Fig. 2:**
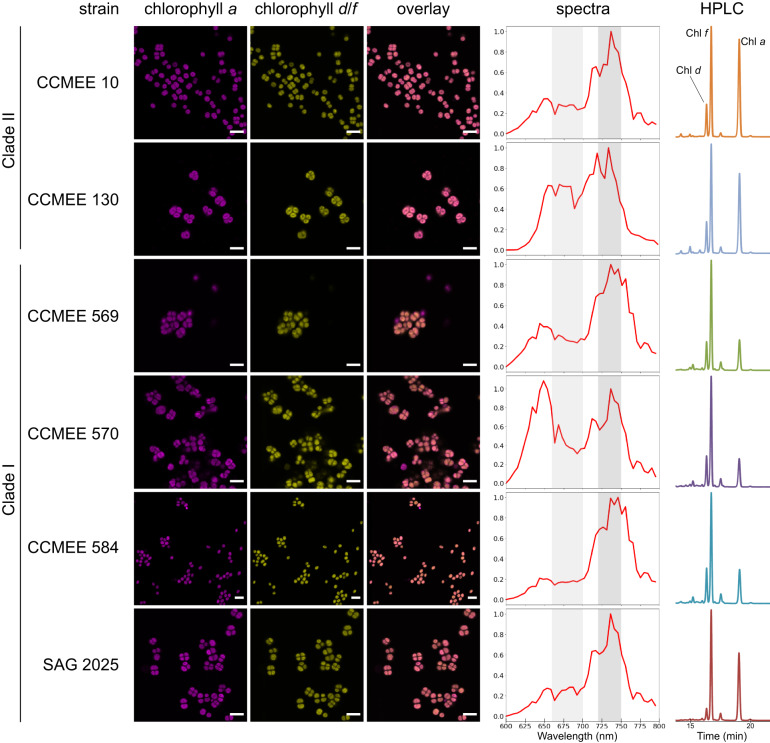
Far-Red Light Photoacclimation in the *Chroococcidiopsidales*. The synthesis of red-shifted chlorophylls and their incorporation into photosystems can be seen in confocal micrographs (left). In addition to florescence emission from chlorophyll *a* and phycobilisomes (magenta, 660–700 nm), there is emission from chlorophyll *f* (yellow, 720–750 nm). Rightmost micrographs mark the overlay of both channels. Excitation, 488 nm. Scale bar, 10 µm. Spectral characteristics can be also observed through a fluorescence emission scan, with the aforementioned channels highlighted (second column from the right). Y-axis: Intensity (a.u.). Composite graph for a minimum of 3 cells. Pigments were extracted and analyzed by HPLC (right). Absorbance traces at 696 nm are shown, indicating the presence of chlorophyll *d* (16.3 min), *f* (16.7 min) and *a* (19.0 min).

To accurately distinguish the FaRLiP cyanobacterial cells from contaminants on a single-cell level, we recorded fluorescence emission spectra using confocal microscopy. All strains surviving in far-red light showed emission peaks that indicated the presence of red-shifted chlorophylls in the photosystems (Fig. 2, or S1 for full results). The chlorophylls involved extend the emission range to 720–750 nm, in addition to the commonly found fluorescence attributed to chlorophyll *a* and phycobilisomes in the range of 640–700 nm at room temperature. This response is specific to far-red light and it does not occur under standard, white-light conditions [24]. As confocal microscopy is unable to identify the specific pigments, HPLC analysis was performed on a subset of 9 strains from groups I and II that were phylogenetically representative and exhibited low contamination levels.

All of the HPLC-tested strains contained chlorophylls *a*, *d*, and *f* when cultured under far-red light (Fig. 2, Table S1). Chromatogram peaks were visually consistent with the published percentages of approx. 90% chl *a*, 9% chl *f* and 1% chl *d* of the chlorophyll content [24]. Chlorophylls *d* and *f* were always observed in HPLC data for samples that showed a red-shifted fluorescence peak under far-red light, and these peaks were always observed for samples containing these red-shifted chlorophylls (Fig. 2). It has been shown that chlorophyll *f* is essential for FaRLiP [24], and the trace pigment chlorophyll *d* has been reported from the majority of strains undergoing this process [27]. However, the literature is divided on the significance of the latter. Some strains appear to lack chlorophyll *d* [21, 30–34], including *Chroococcidiopsis* strains from a subtropical forest [21]. These strains are 98.2-100% similar in 16S rRNA sequence to ours [21, 23]. All of our phylogenetically diverse samples contained chlorophyll *d* (Fig. 1B). This is consistent with previous studies indicating that Photosystem II (PSII) from far-red grown FaRLiP cyanobacteria contain a single chlorophyll *d* pigment in the reaction center, including in the type strain *Chroococcidiopsis thermalis* PCC 7203 [23, 29]. Since the amount of PSII per cell is known to vary depending on growth conditions [78], the detection of chlorophyll *d* might thus be more difficult under certain conditions and explain its apparent absence in some cases. Therefore, we suggest that having one chlorophyll *d* per PSII may be a conserved attribute of all FaRLiP strains. Future studies could consider growth phase in their chlorophyll *d* pigment analyses.

Overall, it is clear that the capacity for FaRLiP is correlated with the phylogenetic lineage. Specifically, within *Chroococcidiopsis sensu stricto* (I), all but one of 24 strains (96%), tested positive for chlorophyll *f* synthesis via multiple methods (marker gene presence, confocal microscopy, HPLC) (Fig. 1). In contrast, in lineage II, the majority of strains (18, or 73%) tested negative for chlorophyll *f* (Fig. S2). Out of 24 desert strains tested, only 3 were positive, belonging to lineage II. This seemingly contradicted our initial expectation that extreme endolithic environments would be enriched in FaRLiP cyanobacteria. Therefore, in order to investigate the connection between FaRLiP and habitat, and whether this is influenced by phylogenetic history, we set out to understand phylogenetic groupings within the *Chroococcidiopsidales*, and the adaptive niches they may be associated with.

### Improved phylogeny of the order Chroococcidiopsidales

**Fig. 3:**
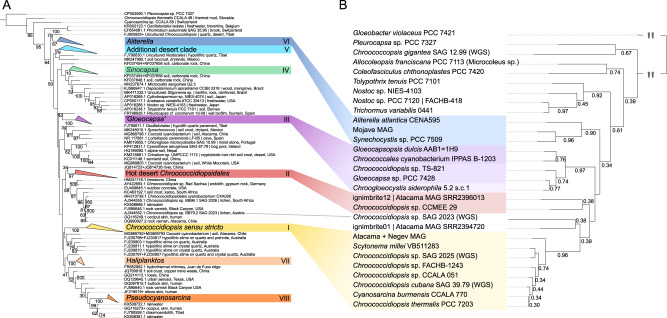
Phylogenetic history of the *Chroococcidiopsidales* order. Phylogenetic trees of the *Chroococcidiopsidales* order based on 16S rRNA genes (**A**) and genome information (**B**). The two trees mirror and complement each other. In (**A**), it can be seen that the genus ‘*Chroococcidiopsis*’ is currently polyphyletic. Strains identified as such actually belong to multiple clades, some of which may be highly supported, but whose relationships with each other is sometimes unclear. Large, highly supported clades and/or clades with type species have been collapsed. Each color represents a genus-level taxon, some with associated genome information (**B**). These taxa include well-defined genera such as *Chroococcidiopsis sensu stricto* (I), *Sinocapsa* (IV), *Aliterella* (VI), *Haliplanktos* (VII) and *Pseudocyanosarcina* (VIII). They also encompass yet-undefined genera lacking a type species—Hot desert *Chroococcidiopsi*dales (II) and ‘Additional desert clade’ (V). Moreover, ‘*Gloeocapsa*’ (III) represents a cluster of taxonomically ambiguous, closely related strains (*Gloeocapsa*, *Gloeocapsopsis*, *Chroogloeocystis* and *Speleotes*). Although showing low support in this tree, the *Sinocapsa* (IV) branch is stable across multiple tree-building methods. The related *Nostocales* are indicated by a gray rectangle (see “Materials and Methods”: Single-gene phylogenies and rooting). The tree was rooted with *Pleurocapsa* sp. PCC 7327, another FaRLiP strain, as an outgroup.

Triggered by the observed divergence in the ApcE2 tree (Fig. 1A), a 16S rRNA gene phylogeny (Fig. 3) and a sequence similarity network (Fig. S3) were built to better understand the evolutionary history of the order *Chroococcidiopsidales*. Trees based on 16S rRNA genes carry limited phylogenetic information and are therefore prone to issues such as unreliable branching patterns, polyphyly, and/or mislabeling. In contrast to them, genome-level phylogenies are less sensitive to noise, but full genomes are available for far fewer strains [36, 40]. This study took advantage of both methods, by building a genome phylogeny (Fig. 3B) and using it to correct and interpret the 16S rRNA gene phylogeny (Fig. 3A).

The resulting *Chroococcidiopsidales* genome tree covered a diversity of lineages, by combining publicly available NCBI data (12 genomes), metagenome bins (3) and locally sequenced strains (2). Of the latter, *Chroococcidiopsis* sp. SAG 2025 was sequenced to the level of a complete, circularized genome (Table S3). Combining our trees with other recent work, showed six highly supported clades (III-VIII) besides the two already mentioned (I-II) (Fig. 3; See Figs. S2, S4–S8 for individual trees) [35, 37–39]. These include five genera, such as *Chroococcidiopsis sensu stricto* (I), *Sinocapsa* (IV), *Aliterella* (previously ‘Cold desert *Chroococcidiopsis*’) (VI), *Haliplanktos* (VII) *Pseudocyanosarcina* (VIII), and the two additional taxa ‘Hot desert *Chroococcidiopsidales*’ (previously ‘Hot desert *Chroococcidiopsis*’) (II) and ‘Additional desert clade’ (V). One extra clade is represented by a monophyletic cluster of genera related to *Gloeocapsa* (III), with >95% similarity between 16S rRNA gene sequences. Because the taxonomy of *Gloeocapsa*-related strains requires revision (Fig. S4), for the purposes of this paper they will be discussed together.

All of these clades, including those yet to be described, are likely equivalent to genera. This can be seen from 16S rRNA gene similarities (>95% within clades) (Table S4) [79], from the shape of D1-D1’ (Fig. S9) and Box-B loops of the rRNA operon’s hypervariable region (Fig. S10), from shared genes (Fig. S11), as well as metadata. For ease of cross-referencing, names used in previous publications were collated (Table S5).

Although it was noted as a possibility from an early landmark paper [80], polyphyly within *Chroococcidiopsis* has been insufficiently acknowledged in the literature [15, 19]. This can lead to biased assumptions in comparative microbiological studies, which can propagate through later research. For example, a model organism used for cryo-EM studies of Photosystem I is *Chroococcidiopsis* sp. TS-821 [81], which we show actually belongs to *‘Gloeocapsa’* (III) (Fig. 3). Awareness of ‘*Chroococcidiopsis*’ diversity grew in recent years through the introduction of genera such as *Aliterella* and *Sinocapsa* [35, 37–39]. Our work highlights two additional genus-level clades (II and V) which have been commonly mislabeled as ‘*Chroococcidiopsis*’. The present phylogeny considers all the data available (16S rRNA genes, genomes, MAGs) to produce a state-of-the-art understanding of evolutionary relationships within the *Chroococcidiopsidales*, and assist further research on this order.

### FaRLiP within the Chroococcidiopsidales is common to generalists, not specialists

**Fig. 4:**
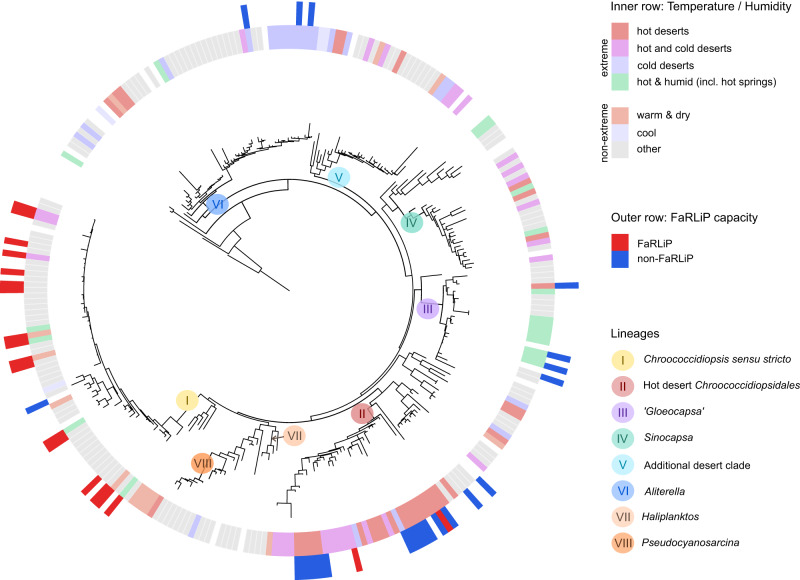
Environmental preferences within *Chroococcidiopsidales* lineages. This figure considers the temperature/humidity of the original sampling sites (inside row), as well as the capacity to utilize far-red light (outside row). Lineages are listed on the tree branches. Lineage II appears to be highly specialized to living in hot deserts. In contrast, *Aliterella* (VI) sequences have often found in cold deserts. *Gloeocapsa* and related strains (III) are characteristic of hot springs, while *Chroococcidiopsis sensu stricto* (I) appears to have a wider distribution. *Chroococcidiopsis sensu stricto* is the largest of the clades recovered, and it is strongly associated with the ability to perform FaRLiP. FaRLiP capacity was assessed by combining experimental and genome data. Strains listed as positive survived under far-red light and / or carried the entire FaRLiP gene cluster. Negative strains did not survive under far-red light, failing the *apcE2* PCR assay, and / or lacked the gene within a complete genome. Tree built with RaxML. An extended version with labels can be accessed at https://itol.embl.de/tree/1301336668200071662129117.

By recovering sampling location data, many clades could be associated with particular (micro)environments, and were therefore defined as ‘specialists’ (Fig. 4). The majority of strains in specialist clades do not appear to have FaRLiP, indicative of gene loss. FaRLiP-lacking clades include ‘*Gloeocapsa*’ (III), which is strongly associated with hot spring or saline environments (Supplementary Text 1), and *Aliterella* (VI) which appears linked to cold deserts, such as polar and alpine regions (Supplementary Text 2).

In specialist lineage II, only 3 out of the 18 tested strains proved capable of FaRLiP. We defined this clade as ‘Hot desert *Chroococcidiopsidales*’. Out of 35 available 16S rRNA gene sequences, 33 were recovered from hypo-/endolithic niches in hot deserts (Fig. 4, S2). Adaptations to extremophilic life have been documented by considerable laboratory work (Supplementary Text 3) [15, 19]. The three FaRLiP strains are CCMEE 10, 12 and 130, with the first two identical in 16S rRNA gene sequences. Out of them, CCMEE 10 has been recently shown to only carry a partial, though functional, FaRLiP cluster, lacking Photosystem I genes [42]. The partial or total loss of FaRLiP genes seems surprising given that these strains are found in far-red light enriched habitats. It is possible that living in stable, extreme environments may result in less competition for light, leading to gene loss. Although gene loss in this phylum has been mainly studied in the highly reduced genomes of marine picocyanobacteria, the environmental stability hypothesized to underpin this genome optimization [36] might also apply to deserts.

The majority of FaRLiP-positive strains in this study fall under the classification of *Chroococcidiopsis sensu stricto* (I) (Fig. 4, Table S6). All but one of the tested strains in this genus were FaRLiP-positive. Considerable support exists for FaRLiP in this group, as the genus included most of the genomes, sequences and strains recovered. Strains within *Chroococcidiopsis sensu stricto* can be defined as generalists, as they appear to inhabit highly diverse environments. They have been found in freshwater, saltwater, as well as in dry soil and on rocks. However, they have only rarely been identified in hyperarid areas, unlike the closely related clade II, Hot desert *Chroococcidiopsidales*. Therefore, in contrast to the generally held view, they appear to be generalists with limited extremotolerance potential, supported by field and laboratory studies [16, 19]. Genome comparisons suggest that, in contrast to related genera, they may carry a conserved group of genes associated with hypoxic conditions (Supplementary Text 4). We hypothesize that these genes could have been co-selected, together with FaRLiP, for living in shaded environments such as soil or microbial mats.

These widespread generalist strains have mostly been underrepresented in discussions of *Chroococcidiopsis* phylogenetic history. Yet, not only do they form their own clade, but it is the largest among the known *Chroococcidiopsidales*. While this might be partially explained by sampling bias (non-extreme environments may be easier to sample), these strains appear to be extremely successful. They live in highly diverse environments, unlike those in extremophile genera. This might be due to higher competition, predation and many additional challenges in these moderate environments that are difficult to discern but shape evolution as much as the more obvious abiotic factors [82].

Not all lineages observed in this study were as straightforward to investigate as *Chroococcidiopsis sensu stricto*. No genomes, and very few strains, were available for clades IV-V and VII-VIII. Therefore, we were unable to assess their FaRLiP capacity. Regarding environments, for *Sinocapsa* (IV) and ‘Additional desert clade’ (V), there were at least sufficient 16S rRNA gene sequences available with associated metadata to suggest that these groups may be extremotolerant (Fig. 4). The latter group included sequences sampled a wide variety of desert environments (Utah, Tibet, Atacama). For groups VII-VIII, there were few sequences available, and no inferences could be made.

This study relied on recovering sampling locations from 16S rRNA datasets to balance out the limited strain availability. It proved particularly valuable for taxonomic groups for which cultured strains are rare, difficult to obtain/grow (as is the case with many polar microbes) or labeled with varying accuracy. It also allowed for correction of previous assumptions about prokaryote dispersal. Likely due to limited data, *Chroococcidiopsis*-related lineages have long been considered to be largely constrained to specific continents [15]. The present study, supported by additional work [12], contradicts this, showing that gene flow occurs frequently. Although nearby sampling locations may share closely related strains (sequences with >99% identity from similar locations were trimmed in our study) [15], this does not scale at higher taxonomic or geographic levels. For example, within a small branch of the *Aliterella* 16S rRNA gene tree, there are sequences from four continents, in no particular pattern. This is especially remarkable for extremophile clades, as deserts have been described functionally as “islands” [15]. Yet widespread geographic dispersal undoubtedly also plays a significant role for generalists, as they encounter diverse environments where survival may involve relying on various acclimation processes, such as FaRLiP.

## Conclusion

*Chroococcidiopsis* has been previously defined as an extremophile cyanobacterium. However, many of the strains known under this name form separate genera. Some of them are specialists adapted to extreme environments such as hot or cold deserts; others are generalists. By combining bioinformatics data mining with large-scale laboratory assays, we have been able to connect genes, genomes, environments and adaptations. FaRLiP has been inherited from a common ancestor, and it remains near-universal in *Chroococcidiopsis sensu stricto*, a generalist genus. However, it has been commonly lost in specialist extremophile genera. It may have been co-selected with genes for hypoxic environments. Chlorophyll *d* was present in all our FaRLiP samples, supporting its predicted significant role in FaRLiP.

### Supplementary information

Supplemental Material

## Data Availability

The expanded version of the large 16S rRNA gene phylogeny can be accessed at iTOL (https://itol.embl.de/tree/1301336668200071662129117) (Legend in Figure S12). Genomic bins for *Chroococcidiopsis* sp. SAG 2025 and *Chroococcopsis gigantea* SAG 12.99 have been uploaded to the WGS database (JAOCNC and JAODIG, respectively). Refined ignimbrite bins and the *Chroococcidiopsis* sp. SAG 2023 genome can be found at figshare (https://figshare.com/projects/Chroococcidiopsis-related_metagenomic_data/149038). Accession numbers for additional sequences are listed in Table S7.
