# Supplementary material for: Common loss of far-red light photoacclimation in cyanobacteria from hot and cold deserts: a case study in the Chroococcidiopsidales
Source: ISME Commun. 2023 Oct 19;3:113. doi: 10.1038/s43705-023-00319-4 (PMC10587186; doi:10.1038/s43705-023-00319-4)
Supplement: Supplementary file 1 — Supplemental Material [file 43705_2023_319_MOESM1_ESM.pdf]

Supplementary information

Strain name	Culture collection[a]	Strain #	*apcE2*	CLSM	HPLC	FRL survival	Env.[b]	Sampling location
Chroococcidiopsis sp.	BCCM/ULC	194					C	Under snow, marble, Antarctica
Chroococcidiopsis sp.	BCCM/ULC	195					C	Cryptoendolith, North side, boulder, Antarctica
Chroococcidiopsis sp.	BCCM/ULC	197					C	Lichen crust (not phycobiont), gneiss scree, Antarctica
C. cubana	CCALA	40					NE	Periphyton, mineral spring, Cuba
C. cubana	CCALA	41					NE	Basin, Cuba
C. cubana	CCALA	42					NE	Stone, mineral spring, Cuba
C. cubana	CCALA	43					NE	Stone, mineral spring, Cuba
C. cubana	CCALA	44					NE	Ditch, Cuba
C. cf cubana [c]	CCALA	45					NE	Dry soil, Pinar del Rio, Cuba
Chroococcidiopsis sp.	CCALA	46					NE	Psammon, Mamaia beach, Romania
C. cf cubana	CCALA	47					NE	Reservoir, periphyton, Czech Republic
C. thermalis	CCALA	48					NE	Cuba
C. thermalis	CCALA	50					NE	Periphyton, thermal spring, Slovakia
Chroococcidiopsis sp.	CCALA	51					NE	Belanske Tatry, Slovakia
Chroococcidiopsis sp.	CCALA	52					NE	Subaerophyte, stone, Yerevan, Armenia
'Cyanosarcina' sp.	CCALA	63					NE	Littoral, lake, Constanta, Romania
'Cyanosarcina' sp.	CCALA	66					NE	Periphyton, mineral water, Slana Voda, Slovakia
Chroococcidiopsis sp.	CCALA	927					NE	Lake Strbske Pleso, Mts. Vysoke Tatry, Slovakia
Chroococcidiopsis sp.	CCMEE	6					H	Cryptoendolith on sandstone, Negev desert, Israel
Chroococcidiopsis sp.	CCMEE	7					H	Cryptoendolith on granite, Negev desert, Israel
Chroococcidiopsis sp.	CCMEE	10					H	Chasmoendolith on granite, Negev desert, Israel
Chroococcidiopsis sp.	CCMEE	12					H	Cryptoendolith on sandstone, Negev desert, Israel
Chroococcidiopsis sp.	CCMEE	19					H	Magnesium calcite, Negev desert, Israel
Chroococcidiopsis sp.	CCMEE	29					H	Cryptoendolith on sandstone, Negev desert, Israel
Chroococcidiopsis sp.	CCMEE	46					H	Hypolith on limestone, Negev desert, Israel
Chroococcidiopsis sp.	CCMEE	53					H	Chasmoendolith on granite, Sinai Desert, Egypt
Chroococcidiopsis sp.	CCMEE	57					H	Chasmoendolith on granite, Sinai Desert, Egypt

Strain name	Culture collection[a]	Strain #	*apcE2*	CLSM	HPLC	FRL survival	Env.[b]	Sampling location
Chroococcidiopsis sp.	CCMEE	64					H	Hypolithic, desert pavement, Sinai Desert, Egypt
Chroococcidiopsis sp.	CCMEE	75					H	Chasmoendolithic on quartz, Death Valley, USA
Chroococcidiopsis sp.	CCMEE	78					HC	Chasmoendolithic on gravel, Mojave Desert, USA
Chroococcidiopsis sp.	CCMEE	88					H	Chasmoendolithic, granite, Sonora Desert, Mexico
Chroococcidiopsis sp.	CCMEE	89					H	Chasmoendolithic, granite, Sonora Desert, Mexico
Chroococcidiopsis sp.	CCMEE	102					H	Chasmoendolithic, granite, Sonora Desert, Mexico
Chroococcidiopsis sp.	CCMEE	130					HC	Cryptoendolithic on sandstone, Canyon Lands, USA
Chroococcidiopsis sp.	CCMEE	171					C	Cryptoendolithic on sandstone, University Valley, Antarctica
Chroococcidiopsis sp.	CCMEE	313					H	Hypolithic, rock, Broken Hill, Australia
Chroococcidiopsis sp.	CCMEE	569					HC	Hypolithic, desert pavement, Gobi desert, Mongolia
Chroococcidiopsis sp.	CCMEE	570					HC	Hypolithic, desert pavement, Gobi desert, Mongolia
Chroococcidiopsis sp.	CCMEE	584					HC	Hypolithic, desert pavement, Gobi desert, Mongolia
C. thermalis [d]	PCC	7203					NE	Soil, Germany
C. cubana [d]	SAG	39.79					NE	Dry soil, Pinar del Rio, Cuba
Chroococcidiopsis sp.	SAG	2023					NE	Phycobiont of lichen *Thyrea pulvinata*, Austria
Chroococcidiopsis sp.	SAG	2024					NE	Phycobiont of lichen *Anema nummularium* var. nummulariellum, Mexico
Chroococcidiopsis sp.	SAG	2025					NE	Endolithic in coral rock, Hawaii
Chroococcidiopsis sp. [e]	SAG	2026					NE	Phycobiont of lichen *Peltula euploca*, South Africa

Table S1. Strains used in the study. Multiple pieces of evidence show their capacity to either acclimate to far-red light (blue) or not (red). '*apcE2*' refers to the *apcE2* marker gene PCR assay; 'CLSM' is confocal laser scanning microscopy. Most strains which tested positive for the marker gene also showed the presence of red-shifted chlorophylls, but a minority (e.g. strains CCALA 43 and 44) did not complete the transition. It is possible that this could be affected by other organisms competing in non-axenic cultures. [a] BCCM/ULC (University of Liège, Belgium), CCALA (Culture Collection of Autotrophic Organisms, Czech Republic), CCMEE (Culture Collection of Microorganisms from Extreme Environments), PCC (Pasteur Culture Collection of Cyanobacteria, France), SAG (Culture Collection of Algae at the University of Göttingen, Germany). [b] Sampling environments were categorized into hot deserts (H), cold deserts (C), hot-and-cold deserts (HC) and non-extreme (NE). [c] *C. cf cubana* CCALA 45 is equivalent to *C. cubana* SAG 39.79. [d] Data for *C. thermalis* PCC 7203 and *C. cubana* SAG 39.79 can be found in previous work [1, 2]. [e] While originally *Chroococcidiopsis* sp. SAG 2026 was tested positive for all markers listed, it was discovered that the lab strain used was a duplicate of *Chroococcidiopsis* sp. SAG 2025. Strain Bü 96.1 (origin of, and equivalent to SAG 2026) was tested in its place and proved negative.

Primers	Sequence (5’ to 3’)	Degeneracy	Length	Target	Reference
f_apcE2M*	CAGAGGATTGCGCGGTGTHATYCCHGAAGATRTNAC	144	36	*apcE2*	[2]
r_apcE2	GGAGCTCTGCAGATATCGCCCGATGRATRTATTCDRY	48	37	*apcE2*	[2]
f_apcE2t*	CAGAGGATTGCGCGGTG	0	17	*apcE2*	[2]
r_apcE2t	GGAGCTCTGCAGATATCGCC	0	20	*apcE2*	[2]
CYA359F	GGGGAATYTTCCGCAATGGG	2	20	16S rRNA	[3]
C_uni_16S	CGGGCGGTGTGTAC	0	16	16S rRNA	[4]
CSIF	GYCACGCCCGAAGTCRTTAC	2	20	ITS	[5]
ULR	CCTCTGTGTGCCTAGGTATC	0	20	ITS	[5, 6]

Table S2. Primers used in the study.

Strain / bin name	Completion (%)	Redundancy (%)	Coverage (x)	Contigs #	Assembly size (bp)	Largest contig (bp)	L50	N50	CDS #	tRNA #	rRNA #	Source
Chroococcidiopsis cubana SAG 39.79	98.59	1.81	70	54	8 257 333	956 021	8	334 641	8 878	45	3	JAVKZF000000000
Chroococcidiopsis sp. SAG 2023	90.77	0.22	22	22	6 130 668	1 444 241	3	818 528	8 098	42	4	figshare
Chroococcidiopsis sp. SAG 2025	98.48	1	150	14	8 100 735	6 664 113	1	6 664 113	8 048	44	6	JAOCNC000000000
Chroococcopsis gigantea SAG 12.99	99.78	0.37	400	8	4 896 497	4 317 808	1	4 317 808	4 647	44	4	JAODIG000000000
ignimbrite01 Atacama MAG SRR2396013	78.37	1.22	280	116	3 106 317	98 448	29	38 171	2 937	29	0	figshare
ignimbrite12 Atacama MAG SRR2394720	85.98	2.07	500	549	4 543 802	50 707	126	11 306	4 113	29	1	figshare

Table S3. Statistics for assembled genomes and metagenome bins. Where possible, these were submitted to NCBI. Low sequencing depth for SAG 2023 resulted in a large number of indels, and as such this genome will be made available on figshare instead, together with the low-completion ignimbrite MAGs. Figshare repository: https://figshare.com/projects/Chroococcidiopsis-related_metagenomic_data/149038.

	Pleurocapsa sp. PCC 7327	*Chroococci-diopsis sensu stricto* (I)	Sister-clade to (I)[a]	Hot desert *Chroococci-diopsidales* (II)	Sister-clade to (II)[a]	*'Gloeocapsa'* (III)	*Sinocapsa* (IV)	Additional desert (V)	*Aliterella* (VI)
Pleurocapsa sp. PCC 7327	N/A								
Chroococcidiopsis sensu stricto (I)	88.6	95.95							
Sister-clade to (I)[a]	89.11	90.16	92.77						
Hot desert *Chroococcidiopsidales* (II)	89.11	90.4	93.02	96.76					
Sister-clade to (II)[a]	88.63	90.29	92.26	94.08	94.76				
'Gloeocapsa' (III)	89.62	89.39	91.72	90.96	90.95	96.18			
Sinocapsa (IV)	89.26	88.09	90.88	90.36	90.15	91.49	95.83		
Additional desert (V)	88.56	88.29	91.48	92.34	91.18	90.53	91.21	95.33	
Aliterella (VI)	89.05	88.51	91.34	91.45	90.72	90.88	91.11	92.26	95.6

Table S4. Average similarity of 16S rRNA gene sequences between different *Chroococcidiopsidales* clades under study, or within the same clade. For the outgroup (*Pleurocapsa*), only one sequence was used (*Pleurocapsa* sp. PCC 7327). Clades numbered as shown in Figures 3-4. [a]Two additional groups were used in this analysis, which represent the sister-clades to *Chroococcidiopsis sensu stricto* (I) and Hot desert *Chroococcidiopsidales* (II) in these figures, with low support values. Within clades, any two sequences share on average >95% similarity (blue). Between clades, this average typically falls to 90-92% (yellow) or even <90% (red) for highly divergent groups. This supports the assignment of these clades as separate genera [7].

Lineages in the current study [a]	Jung *et al.*, 2021 [8]	Fagliarone *et al.*, 2017 [9]	Bahl *et al.*, 2011 [10]	Pointing *et al.*, 2007 [b] [11]	Fewer, Friedl & Büdel, 2002 [12]
***Chroococcidiopsis sensu stricto* (I)**	*Chroococcidiopsis sensu stricto* + *Chroocccidiopsis* outgroup	*Chroococcidiopsis* (II)	Hot desert *Chroococcidiopsis* (clade 2)		*Chroococcidiopsis*
**Sister-clade to (I)**			Hot desert *Chroococcidiopsis* (clade 2)		
**Hot desert *Chroococcidiopsidales* (II)**		Hot- and cold-dry desert *Chroococcidiopsis* (IA)	Hot desert *Chroococcidiopsis* (clade 1)	Hot-dry *Chroococcidiopsis*	*Chroococcidiopsis* (hot and cold desert strains)
**Sister-clade to (II)**	Undefined genus	Unspecified (sister-clade to IA)		Unspecified (sister-clade to 'Hot-dry *Chroococcidiopsis*')	*Chroococcidiopsis*
**‘*Gloeocapsa*’ (III)**	*Gloeocapsa* + *Chroogloeocystis* + *Gloeocapsopsis*				
***Sinocapsa* (IV)**	*Sinocapsa*			Common *Chroococcidiopsis*	
**Additional desert clade (V)**			Cold desert *Chroooccidiopsis*	Hot-wet *Chroococcidiopsis*	
***Aliterella* (VI)**	*Aliterella*	Cold-dry desert *Chroococcidiopsis* (IB)	Cold desert *Chroooccidiopsis*		

Table S5. *Chroococcidiopsidales* clades in scientific literature. [a] “Sister-clade to (I)” and “Sister-clade to (II)” appear to be sister clades to *Chroococcidiopsis sensu stricto* (I) and Hot desert *Chroococcidiopsidales* (II), respectively, and may be part of the same genera. They were listed separately due to low bootstrap support at the branching point. [b] The hot-wet *Chroococcidiopsis* clade in Pointing *et al.*, 2007 might be equivalent to the ‘Additional desert clade’ (97% similarity between some sequences), but the short sequences make it difficult to confirm.

Genus	Strains tested (#)	FaRLiP-positive (#)	FaRLiP-positive (%)
***Chroococcidiopsis sensu stricto* (I)**	24	23	96
**Hot desert *Chroococcidiopsidales* (II) [a]**	18	3	27
***'Gloeocapsa'* (III)**	4 (6)	0	0
***Aliterella* (VI)**	3 (4)	0	0
**Other [b]**	2	0	0

Table S6. Summary of FaRLiP distribution in the *Chroococcidiopsidales*. The numbers include strains tested in this study, in a previous study [13], and those with a complete genome available where the presence/absence of the FaRLiP cluster can be confirmed. Numbers in parentheses also consider fragmented genomes. [a] Two of the positive Hot desert *Chroococcidiopsidales* strains were identical in 16S rRNA. [b] Other strains tested include SAG 2023 and SAG 2026.

Strain name	Culture collection[a]	Strain #	*apcE2*	16S rRNA gene	rRNA ITS
Chroococcidiopsis sp.	BCCM/ULC	194	N/A	OK576201	OR242221
Chroococcidiopsis sp.	BCCM/ULC	195	N/A	OK576202	OR242222
Chroococcidiopsis sp.	BCCM/ULC	197	N/A	OK576203	OR242223
C. cubana	CCALA	40	OR233339	AB074506	
C. cubana	CCALA	41	OR233340	MK484709	
C. cubana	CCALA	42	OR233341	MK484707	
C. cubana	CCALA	43	OR233342	MH208394	
C. cubana	CCALA	44	not sequenced	MH208395	
C. cf cubana	CCALA	45	OR233343	MH208396	
Chroococcidiopsis sp.	CCALA	46	OR233344	MK484710	
C. cf cubana	CCALA	47	OR233345	MH208398	
C. thermalis	CCALA	48	OR233346	OR241450	
C. thermalis	CCALA	49	not tested	OR241468	
C. thermalis	CCALA	50	OR233347	MH208399	
Chroococcidiopsis sp.	CCALA	51	OR233348	MH208404	
Chroococcidiopsis sp.	CCALA	52	OR233349	MH208400	
Chroococcidiopsis sp.	CCALA	58	not tested	OR241467	
‘Cyanosarcina’ sp.	CCALA	63	OR233350	OR257827	
‘Cyanosarcina’ sp.	CCALA	66	OR233351	OR257828	
Chroococcidiopsis sp.	CCALA	927	OR233352	OR241451	
Chroococcidiopsis sp.	CCMEE	6	N/A	OR241452	
Chroococcidiopsis sp.	CCMEE	7	N/A	OR241453	
Chroococcidiopsis sp.	CCMEE	10	OR233356, OM156474	OR241454	OR413562
Chroococcidiopsis sp.	CCMEE	12	not sequenced	OR241455	
Chroococcidiopsis sp.	CCMEE	19	N/A	OR241456	
Chroococcidiopsis sp.	CCMEE	29	N/A	CP083761	
Chroococcidiopsis sp.	CCMEE	46	N/A	OR241457	
Chroococcidiopsis sp.	CCMEE	53	N/A	OR241458	
Aliterella sp.	CCMEE	71	not tested	OR241469	
Chroococcidiopsis sp.	CCMEE	75	N/A	OR241459	
Chroococcidiopsis sp.	CCMEE	78	N/A	OR241460	

Chroococcidiopsis sp.	CCMEE	88	N/A	OR241461	OR242215, OR242216
Chroococcidiopsis sp.	CCMEE	89	N/A	OR241462	
Chroococcidiopsis sp.	CCMEE	102	N/A	OR241463	
Chroococcidiopsis sp.	CCMEE	130	OR233357	OR241464	OR242217, OR242218
Chroococcidiopsis sp.	CCMEE	171	N/A	JF810071	
Chroococcidiopsis sp.	CCMEE	313	N/A	OR241465	OR242219, OR242220
Chroococcidiopsis sp.	CCMEE	569	OR233353	MF289558	
Chroococcidiopsis sp.	CCMEE	570	OR233354	OR241466	
Chroococcidiopsis sp.	CCMEE	584	OR233355	AY301004	
C. thermalis	PCC	7203	CP003597	CP003597	
C. cubana	SAG	39.79	GCA_003991895	GCA_003991895	
Chroococcidiopsis sp.	SAG	2023	N/A	AJ344552	
Chroococcidiopsis sp.	SAG	2024	N/A	AJ344553	
Chroococcidiopsis sp.	SAG	2025	OR233358	AM709635	
Chroococcidiopsis sp.	SAG	2026	N/A	AJ344555	

Table S7. NCBI accession numbers. The entries listed in red have been submitted to the NCBI database in the course of this study. If the sequences recovered proved identical to/shorter than existing data, they were not submitted. Some strains (CCMEE 88, 130, 313) carry two versions of the ITS, with the longer variant including tRNA-Ala and tRNA-Ile genes.

NCBI accession number	Label	Revised taxonomic unit	Maximum identity (%)	Reference
DQ058858.1	*Chroococcidiopsis* sp. SCyano33	Unclear. Likely a novel lineage related to *Pleurocapsales* / *Spirulinales*	91	[14]
AJ344557.1	*Chroococcidiopsis* sp. PCC 6712	*Pleurocapsales*	95	[12]
MG022133.1	*Chroococcidiopsis* sp. QUCCCM26	*Pleurocapsales* (likely *Xenococcaceae*)	97	[15]
JF810077.1, JF810078.1, JF810079.1	*Chroococcidiopsis* sp. CCMP3184/5/7	*Pleurocapsales*	96	[16]
JF810074.1, AJ344556.1	*Chroococcidiopsis* sp. CCMP2623 / CCMP1489	*Pleurocapsales*	96	[12, 16]
JF810076.1	*Chroococcidiopsis* sp. CCMP2733	*Pleurocapsales*	97	[16]
HQ832924.1	*Chroococcidiopsis* sp. LEGE 06174	*Pleurocapsales*	99	
JF810070.1	*Chroococcidiopsis* sp. CCMP2116	Unclear. Likely related to early-branching filamentous cyanobacteria	97	[16]
KU321248.1	*Chroococcidiopsis thermalis* NGPC10ME	*Synechoccaceae*	100	n/a
DQ914866.2	*Chroococcidiopsis* sp. CC4	uncertain	94	[11]
	Cyanosarcina sp. CCALA 58	uncertain	100	n/a
	Chroococcidiopsis thermalis CCALA 49	probably *Pleurocapsales*	99	n/a

Table S8. Revised taxonomic assignment of strains labeled as *Chroococcidiopsis* whose 16S rRNA sequences are very different from *Chroococcidiopsis thermalis* PCC 7203 (<90% similarity). They were recovered by the use of standard filters in web BLAST. Maximum identity represents the highest identity score when the sequence is queried against the non-redundant NCBI database. Sequences labeled as ‘uncultured’ were not considered.

Group	Classification	Sub-classification	NCBI code	Putative function
***Chroococcidiopsis* (I)**	Metabolism	Glycerolipid metabolism	WP_015154930.1	lysophospholipase / monoacylglycerol lipase
***Chroococcidiopsis* (I)**	Metabolism	Fatty acid metabolism	WP_015154535.1	fatty acid desaturase
***Chroococcidiopsis* (I)**	Metabolism	Amino acids	WP_015152497.1, WP_015155071.1, WP_015153197.1, WP_015155764.1, WP_015155967.1	
***Chroococcidiopsis* (I)**	Metabolism	Citrate cycle	WP_015154032.1	putative malate dehydrogenase (photosynthesis - > aminoacid)
***Chroococcidiopsis* (I)**	Metabolism	Galactose metabolism	WP_015154720.1	phosphoglucomutase (connects to purine metabolism)
***Chroococcidiopsis* (I)**	Metabolism	Fructose metabolism	WP_015157347.1	class II fructose-bisphosphate aldolase + uncertain
***Chroococcidiopsis* (I)**	Metabolism	Sulfur metabolism	WP_015153197.1*, WP_015157392.1, WP_015153655.1*	Includes what might be a sulfite oxidase. *'Gloeocapsa'* version very low homology
***Chroococcidiopsis* (I)**	Metabolism	Carbon fixation pathways in prokaryotes	AFY89799.1	*nifJ* (pyruvate:ferredoxin (flavodoxin) oxidoreductase)
***Chroococcidiopsis* (I)**	Carbon metabolism		WP_015154223.1, WP_015152226.1, WP_015152497.1, WP_015156403.1*, WP_015155967.1, WP_015152151.1	glucose - > gluconate; putative rare gluconolactonase
***Chroococcidiopsis* (I)**	Carbon metabolism	Starch and sucrose metabolism	WP_015155195.1, WP_015154222.1	
***Chroococcidiopsis* (I)**	Energy Metabolism	Photosynthesis - antenna proteins	WP_015153831.1, WP_051033248.1	*apcF2*, putative PEC synthase
***Chroococcidiopsis* (I)**	Energy Metabolism	Oxidative phosphorilation	WP_015153233.1	Hox bidirectional hydrogenase; also present in a *'Gloeocapsa'*
***Chroococcidiopsis* (I)**	Environmental Information Processing	Two-component system	WP_015152193.1, WP_015155951.1	trypsin-like peptidase (for misfolded proteins?)
***Chroococcidiopsis* (I)**	Environmental Information Processing	Two-component system	WP_015152713.1, WP_015154564.1, WP_015152711.1, WP_015152712.1, WP_015152714.1, WP_015152713.1, WP_015154564.1,	pathways associated with chemotaxis, although it is not known to be motile
***Chroococcidiopsis* (I)**	Environmental Information Processing	ABC Transporters	AFY90866.1, WP_015157404.1, AFY88735.1, WP_015152723.1, WP_015153654.1, WP_015152640.1	Fe(3+) ABC transporter / low iron response?, phosphate
***Chroococcidiopsis* (I)**	Metabolism	Nucleotide metabolism	WP_015154001.1, WP_015154002.1, WP_015153713.1	Includes xanthine dehydrogenase
***Chroococcidiopsis* (I)**	Metabolism	Glutathione metabolism	WP_015152913.1*	spermidine synthase
***Chroococcidiopsis* (I)**	Metabolism	O-Antigen nucleotide sugar biosynthesis	WP_015155421.1, WP_015153184.1, WP_015156730.1, WP_015156319.1, WP_015156749.1	
***Chroococcidiopsis* (I)**	Metabolism	Folate biosynthesis	WP_015153030.1, WP_015155113.1, WP_015154210.1*	putative molybdopterin factor
**Hot desert *Chroococcidiopsidales* (II)**	Environmental Information Processing	ABC Transporters		molybdate transporter
**Hot desert *Chroococcidiopsidales* (II)**	Metabolism of cofactors and vitamins	Ubiquinone and other terpenoid-quinone biosynthesis		putative oxidoreductase, phytoene desaturase (also in *'Gloeocapsa'*)

Group	Classification	Sub-classification	NCBI code	Putative function
**Hot desert *Chroococcidiopsidales* (II)**	Glycan biosynthesis and metabolism	Lipopolysaccharide biosynthesis		multiple glycotransferases and a decaprenyl-phosphate phosphoribosyltransferase (associated with cell wall biosynthesis)
**Hot desert *Chroococcidiopsidales* (II)**				endonuclease
**Hot desert *Chroococcidiopsidales* (II)**	Biofilm formation		*	beta-galactosidase / beta-xylanase
**Hot desert *Chroococcidiopsidales* (II)**	Biofilm formation		*	beta-galactosidase / beta-xylanase
***'Gloeocapsa'* (III)**	Metabolism	Lipid metabolism	WP_015188571.1	heme peroxidase
***'Gloeocapsa'* (III)**	Metabolism	Fatty acid biosynthesis/Lipid metabolism	WP_015190369.1*, AFZ29742.1*, WP_015190083.1, WP_015190271.1, WP_015191117.1*, WP_015186606.1	dehydrogenase, acyl-CoA desaturase, oxidoreductase, beta ketoacyl synthase
***'Gloeocapsa'* (III)**	Metabolism	Sulfur metabolism	WP_015190338.1, AFZ32478.1	2 genes in assimilatory sulfur reduction
***'Gloeocapsa'* (III)**	Energy Metabolism	Oxidative phosphorilation	WP_015189641.1	NAD(P)/FAD-dependent oxidoreductase
***'Gloeocapsa'* (III)**	Metabolism	Amino acids	WP_015187358.1	glutaminase
***'Gloeocapsa'* (III)**	Metabolism	Starch and sucrose metabolism	WP_015188270.1	trehalase
***'Gloeocapsa'* (III)**	Metabolism	Starch and sucrose metabolism	WP_015189877.1, WP_015188541.1, WP_015189884.1, AFZ33259.1,	genes for cellulose synthesis/degradation (cellobiose, cellodextrin...)
***'Gloeocapsa'* (III)**	Metabolism	Amino acids	WP_015189155.1, WP_015190337.1	aminotransferase, cysteine synthase
***'Gloeocapsa'* (III)**	Metabolism	Carbon metabolism	WP_015191119.1	methylenetetrahydrofolate reductase
***'Gloeocapsa'* (III)**	Metabolism	Purine and pyrimidine metabolism	WP_015189457.1, WP_015190554.1*, WP_015189032.1*, WP_015190548.1, WP_015189620.1, AFZ31161.1	
***'Gloeocapsa'* (III)**	Metabolism of other amino acids	Selenocompound metabolism?	WP_015190927.1	FAD-dependent oxidoreductase
***'Gloeocapsa'* (III)**	Metabolism of cofactors and vitamins	Folate biosynthesis	WP_015186902.1, WP_015190264.1*, WP_015191119.1	2 alkaline phosphatases, methylenetetrahydrofolate reductase
***'Gloeocapsa'* (III)**	Metabolism of terpenoids and polyketides	Carotenoid biosynthesis	WP_015189359, AFZ31457.1	2 genes for ketocarotenoids, esp. canthaxanthin; also in CCMEE 029
***'Gloeocapsa'* (III)**	Metabolism of terpenoids and polyketides	Phenylpropanoid biosynthesis	WP_015190920*	cellobiase (mislabeled?)
***'Gloeocapsa'* (III)**	Metabolism of terpenoids and polyketides		WP_015188788.1	unknown
***'Gloeocapsa'* (III)**			WP_015190547.1	amidase
***'Gloeocapsa'* (III)**			WP_015189036.1, WP_015189038.1	cyanuric acid amidohydrolase
***'Gloeocapsa'* (III)**			WP_015190548.1	dihydropyrimidinase

Group	Classification	Sub-classification	NCBI code	Putative function
***'Gloeocapsa'* (III)**			WP_015328641.1, WP_015328622.1, WP_015328623.1, WP_015328624.1	base excision repair, polymerase III components (also in *Cyanosarcina burmensis*)
***'Gloeocapsa'* (III)**	Environmental Information Processing	ABC Transporters	WP_015188723.1, WP_015188726.1, WP_041919526.1, WP_015191224.1, WP_155824104.1	phosphate and phosphonate transporters
***'Gloeocapsa'* (III)**		Cyanoamino acid metabolism	WP_015190920.1*	glycosylhydrolase
***Aliterella* (VI)**	Environmental Information Processing	ABC Transporters	WP_045053158.1, WP_082065291.1, WP_045053159.1, WP_052672235.1	4 genes phosphate transporter (also in *C. cubana*)
***Aliterella* (VI)**	Environmental Information Processing	Two-component system	KJH72825.1	histidine kinase - potential copper sensor
***Aliterella* (VI)**	Lipid metabolism		WP_045053715.1*, WP_052672397.1, KJH71567.1	acyl-CoA desaturase, lipid kinase, lipooxygenase (potentially introduces kink in backbone)
***Aliterella* (VI)**	Lipid metabolism	Glycerophospholipid metabolism	WP_045053716*	
***Aliterella* (VI)**	Lipid metabolism	Fatty acid biosynthesis	WP_045054837.1	acyl-CoA desaturase (found in a *'Gloeocapsa'*)
***Aliterella* (VI)**	Metabolism	Nitrogen metabolism	WP_045052789.1, WP_045052790.1, WP_144405642.1, WP_045056412.1*	small and large nitrite reductase component (dissimilatory); gene from assimilatory pathway shared with *'Gloeocapsa'*, nitric oxide synthase oxygenase (shared with *C. cubana*)
***Aliterella* (VI)**	Metabolism	Degradation of aromatic compounds	WP_045054871.1	
***Aliterella* (VI)**	Metabolism of cofactors and vitamins	Prodigiosin (mislabeled)	WP_045056679.1*	3-ketoacyl-ACP reductase

Table S9. A subset of clade-specific genes highlighted by OrthoVenn2. Putative pathways were identified with BlastKOALA and KofamKOALA on the KEGG webserver. Putative functions were identified with the above in conjunction with standard BLAST. For the Hot desert *Chroococcidiopsidales* clade (II), there was no published genome available, only a metagenomic bin. As such, the genes in question are listed in Table S10. Asterisks mark genes which have few to no homologues in other cyanobacteria (as judged from BLAST output).

Code	Sequence	Putative function
ignimbrite12_meta<br>|FLMEBPDB_02256	MSWHPFILSLQVTVVATVLILVVGMGLAIVLARSRFPGQLVLETLLNLPL<br>VLPPSVVGYYLLLVLGRGSPLVELLGIRILFTWQAAAIASAVVGLPLMVE<br>SARAAIANVNPELESAARTLGSSEAEVLWRVTLPLARQGILAGLLLGIAR<br>ALGEFGATLMVAGNIPGRTQTLPLAIYDAVQNQQYALANLMVLVMTTLAF<br>VLVWWVRRLERSKGLLSVEHQSKLKNETFSGHSEATP	molybdate transporter
ignimbrite12_meta<br>|FLMEBPDB_04185	MTKVLIVYTTSTGNTKRMAEAVAEGARSVAGAAVTLKEAQLATMDDVRAC<br>DALIMGSPMRHRTADSRIKKFIEDVCEVLWLSDEMVGKVGGVFTVGGGYG<br>DCGAGCELAQLGLLGAFAAGGMILLTYSPELNSGDSRLKQQPQSQTVLRR<br>LT	oxidoreductase
ignimbrite12_meta<br>|FLMEBPDB_00296	MLKQIAIVGAGPGGLATAMRLASQGYTVQIFEAADRVGGRMRGFEDGPYA<br>FDTGPTILQLPRVYEELFAESGLKLADYIHFKQLNPNTRLRFWDDSRLDL<br>TSDLDNFKAQLALMRPDLPEAFDRWYTEHIHKNEVGYKPYLGSPVRSVLG<br>YLRPKEIAAALPLRPWESLYQHFWRFFKDERLVYALSYPSKYLGMHPTVC<br>SSIFSLIPFLEFADGVWHPEGGFRALAQALAKAAMDLGVQIHLNCPVRQV<br>WIEGKHACGVELASGERVSADAVVVNADFGYAVRHLLPEPARDRYTDQIS<br>KMRFSCSTFMLYLGINRRYEDLPHHQLYLSEHIRRRERPWVDDSALDEED<br>PPFYVCNPTIVDPSNAPDGHSTLFVLVPIPNTSYGVDWAAKQQGYRDLII<br>QRMSLLGFEDVEKHIVRETCYTARTWQDDYYVHLGAVFNLSHNWSQLGPF<br>RPPIRSDSIRRLYWIGGAVHPGSGLLTILEAARSAAVFIGEDLAMLCRL	phytoene desaturase (carotenoid biosynthesis)
ignimbrite12_meta<br>|FLMEBPDB_00667	MKNLKVAAYITAYEDYRAVEACVMAVKNQSVKVEKIFIVDNSTRHRVVLS<br>NYEDVIIDSHPENLGVSGGLRIGLEWAFQQNYDFLWTFDQDSIPASNCLE<br>ILLKQYINLSQYNYKIGIIAPTPIDARTNTVVEGAVFDRDRFIGRKHNSQ<br>AQSYECDAPITSGSLISIAAAKTLSLPRTDLFIDSVDHDYGMRLKQKGFH<br>NLIVPQAILHHQFGNPIKVNLLKRERIVQNYSALRHYYICRNQTYLDTRY<br>AQGWYRLTSCIWRMKFMLHTIVLIWLCDPKDKPQKSWACLLGTFHGLQGK<br>LGKTW	glycosyltransferase
ignimbrite12_meta<br>|FLMEBPDB_02326	MCETIAYRVAAYITAYQDLEALDKCITAIERQSYPVQEIFIVDNSITELV<br>SQTRYRNTVVEFHPENIGVAGGLKIGIRWAIEKEYDFLWLFDQDSEPSYD<br>LLEKLLFKHQGLSKTGAKVGIIAPVIFDINTNQEIPGYVFRNYKFVPVVS<br>YEERQDFYRCDGVITSGSLVDLSIAKGVELPREDFFLDAVDYAYCMNFRN<br>KGYEIVVVKNTIMKHRLANYSKVKVRLIKDIKEVITFICSPSRYYYACRN<br>HTFFETRTSPKRILYRTFIYRIKFLIRMIERIVHYEPDLVLLKIWACILG<br>TFDGFRGKLGKTWQ	glycosyltransferase
ignimbrite12_meta<br>|FLMEBPDB_02315	MLEQAQRPSIGAVGALLLYPDDTIQHAGVVAGVAGVANHSHKHYKYGSPG<br>YFNHINTVNNFSAVTAACLMCRRDVFEEVGGFEEDLAVAFNDVDFCFKLV<br>EKGYRNIYLPHVVLYHHESKSRGSEDAAEMPTRFIKEVEHTQSKWKNIIK<br>NDPCYNPNLTRQRIDYSIEG	glycosyltransferase
ignimbrite12_meta<br>|FLMEBPDB_02822	MNNSWQKDYPLQDLTEEGLDENHSLRKMLRLVGENKRVIDFGCATGYFSQ<br>LLAKKGCRVTGVELNPDAAKFAEQYCEQVIVADLDLVPLAEILPGHTFDV<br>AIFGDILEHLRDPWKILKETQQLLNSEGYVVASLPNIAHGAIRLALLQGR<br>FEYTALGILDNTHLRFFTRKTVEDLFERSGYFLDIIDRTKLSVFSETPLI<br>PQIDKNVFNREIVKQIEQDEEAETFQFILRSFPKTPEGKYAALNERYSNL<br>VQLERSLRQASLTQSQLQQTQAQLEQLQSQLQQTQAELERSLRQASLTQA<br>QLQHTQAELQRSQATITSMQTSKFWKLRTKWFNLRQRSKPFNLIEKMYSF<br>VRQLRIKFFYSKKELT	glycosyltransferase / methyltransferase
ignimbrite12_meta<br>|FLMEBPDB_05736	MLEQAQRPSIGAVGALLLYPDDTIQHAGVVAGIGGVAGHSHKHYKYGSSG<br>YFNHINTVNNFSAVTAACLMCRRNVFEEVGGFEEELPDNFNDVDFCFKLV<br>EKGYRNIYLPHVVLYHYESKSRGNDYTGERLNRFIKEVEYIQRRWKKIIK<br>NPPCYNLNLTREREDYSIEM	glycosyltransferase
ignimbrite12_meta<br>|FLMEBPDB_02719	MTSITKISHPYYLLKAMRPHHWVKNLVVFAAPLFALQLDATSVLLASIAF<br>IVFSLTASSFYLINDVADVKADRQHPIKCKRPIAAGLVAIPVALLTAVTC<br>LAGSLLISFSITPWLGISVGTYALVQAGYNVGLKHEPIIDILCISAGFVL<br>RALGGAAATAVPVSDWFVLCVGLLAFYLGIEKRKAELKSLGSKGTTRRVL<br>KQYTLPWLNRMESVVTASTLMSYSLWAIDGAKSHWMLATIPFVTYGIFKY<br>QALSEQGEGEAPEITLFRSNQLLLTVVLWALTSILILSLS	decaprenyl-phosphate phosphoribosyl-transferase
ignimbrite12_meta<br>|FLMEBPDB_02340	MKKRLMITKLPAKFLEENEDIEARLTQAAKVVLNLAQEESRRLQHKVIGT<br>EQILLGLLAEGTGPAAQVLMAKGIDLNKTRSEVERMLSVFSAVEASEIPF<br>SPTAKQSLKLSLEAAQELGHGDIDTGHLLLGLIHQGRQGEGVAVWILRNF<br>NVNLQKLEQQILEHLNNSRLIETDQSDWTTSNAALSNSQLPDSIASEISA<br>RLIFWLVSWVYPRKLGHIFSRRGFELPNKGNFVLGISFMPSKVFKLNPPS<br>YIGIVPDLAVEIKSGNQQLLSLQAKIQHLLSLGLLVGVLIDPDDRNVIIY<br>RANGEVTILTDGDMLSIPELLPGWQLAVSDLWLSEVD	endonuclease
ignimbrite12_meta<br>|FLMEBPDB_00158<br>*	MILQVIHSKRVRTKRRTKKRSNPDRPKHWIILFLLFALGNIVVFKGISFA<br>QRVYLEPPLAAVPANLFGMHIHRIAKTTPWPTVPFTTWRLWDAYVAWPNL<br>EPKKGEWHFEVLDKYLNLADKHHVEVLLPLGLSPAWASDRPTERSAYSPG<br>FAAEPRNIEDWRNYVRTVATRYKGRIRYYEIWNEPNLQQFYSGTVGQMEM<br>LSREAYLILKEIDPSIIVVSPSATGDDTGPSWLEKYLRRGGGAYADVIGY	beta-galactosidase / beta-xylanase

Code	Sequence	Putative function
	HFYVTPKPPEAIVPLVRKVQQIMARHGVSNKPLWNTEAGWAIANSSGSVD PKQVGFPENMKVLTADEAAAYVARSLILAWATGLKRFYWYAWDNQVMGLT EVDGKTVKLPARAYAETQKWLVGAKMSECKSNTQKTWICRLTRSGGYTAW IVWNPERKLIFKLPKTWRVHHVRNLAGARHDLVDLDRLEIGQSPLLLQRS AL	
ignimbrite12_meta |FLMEBPDB_02304 *	MAKVWRYGTLTVAVILLLMLSCTQGLPKFVQSQQLLSVSKPPTSDIPASL FNLHVINLKYGATWPSIPFHGWRSFYADWASLEPKKGEWHFEYLDGEVSL AQKHRVEMMLVLQTTPTWASARPKEKGCCTPDAPKGNTAEASNIENWRNY VRQVATRYKGRVHYYELWNEPNVERFYSGTVKQLVLLNRAAYQVLKEVDP TITVISSAMSPYGDHLRYFEDYLAQGGSKYADVIGYHFYVAPKPPEAMLP LIQQVQALMAKYGLADKSLWNTETGWRIINRDKNINYEEWAGNPLSSGDA SAYIARSYLLSWVMGVERLYWYAWGHRSMGLTDYDARTPKPVTTAYTEVR NWLIGAQMTSCELDKQKTWVCQLKRDRGYLAWIVWNPDKSLLFNLPRTWG VQQTRDLSGKKRKLLQANQVKISPSPLLLERVR	beta-galactosidase / beta-xylanase

Table S10. Sequences specific to clade II (Hot desert *Chroococcidiopsidales*), recovered from the previously-published ignimbrite12 metagenome. Putative functions were identified with BlastKOALA/ KofamKOALA and BLAST.

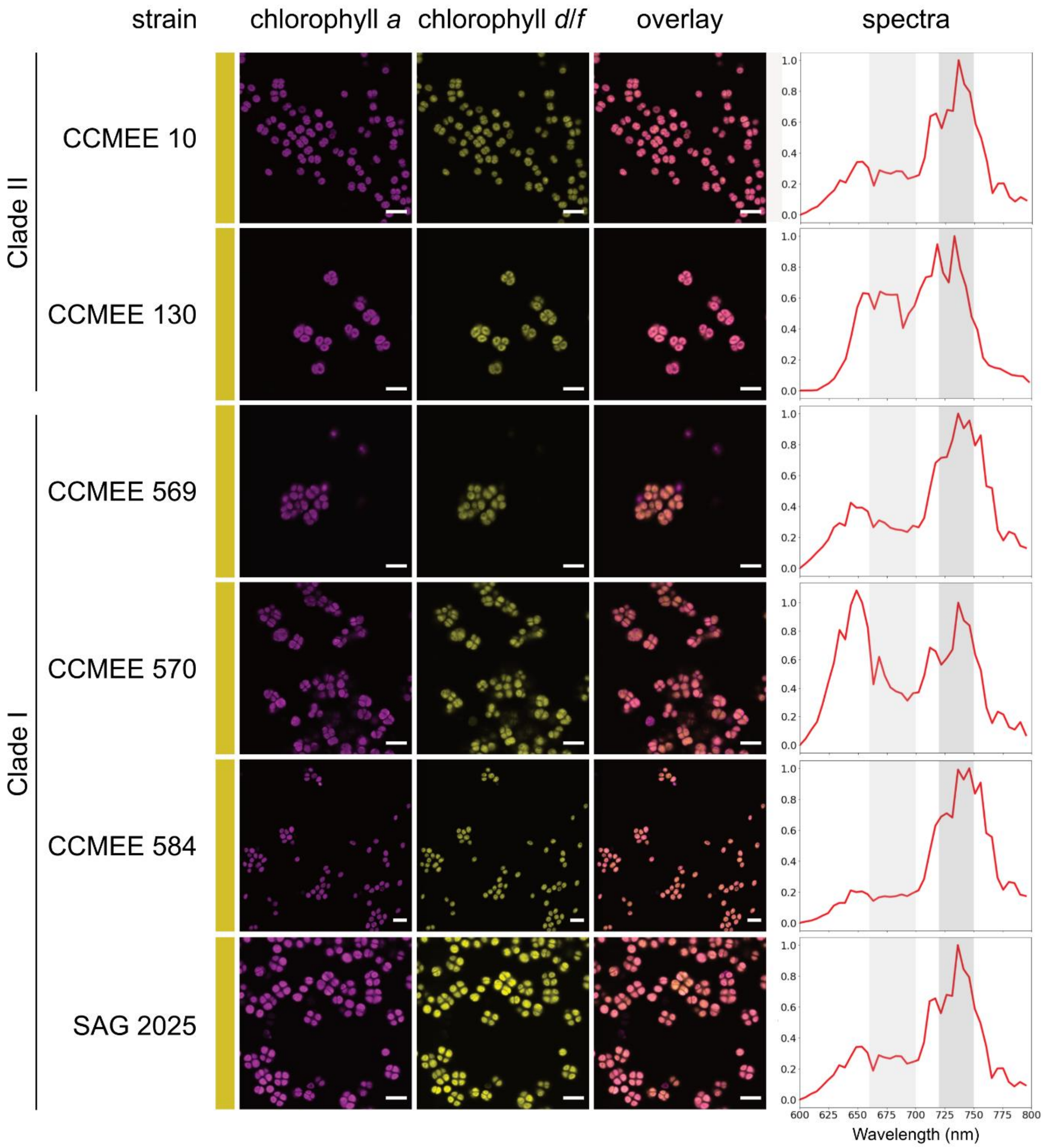

Figure S1. Capacity for FaRLiP in *Chroococcidiopsis*-like cyanobacteria *(figure continues below)*

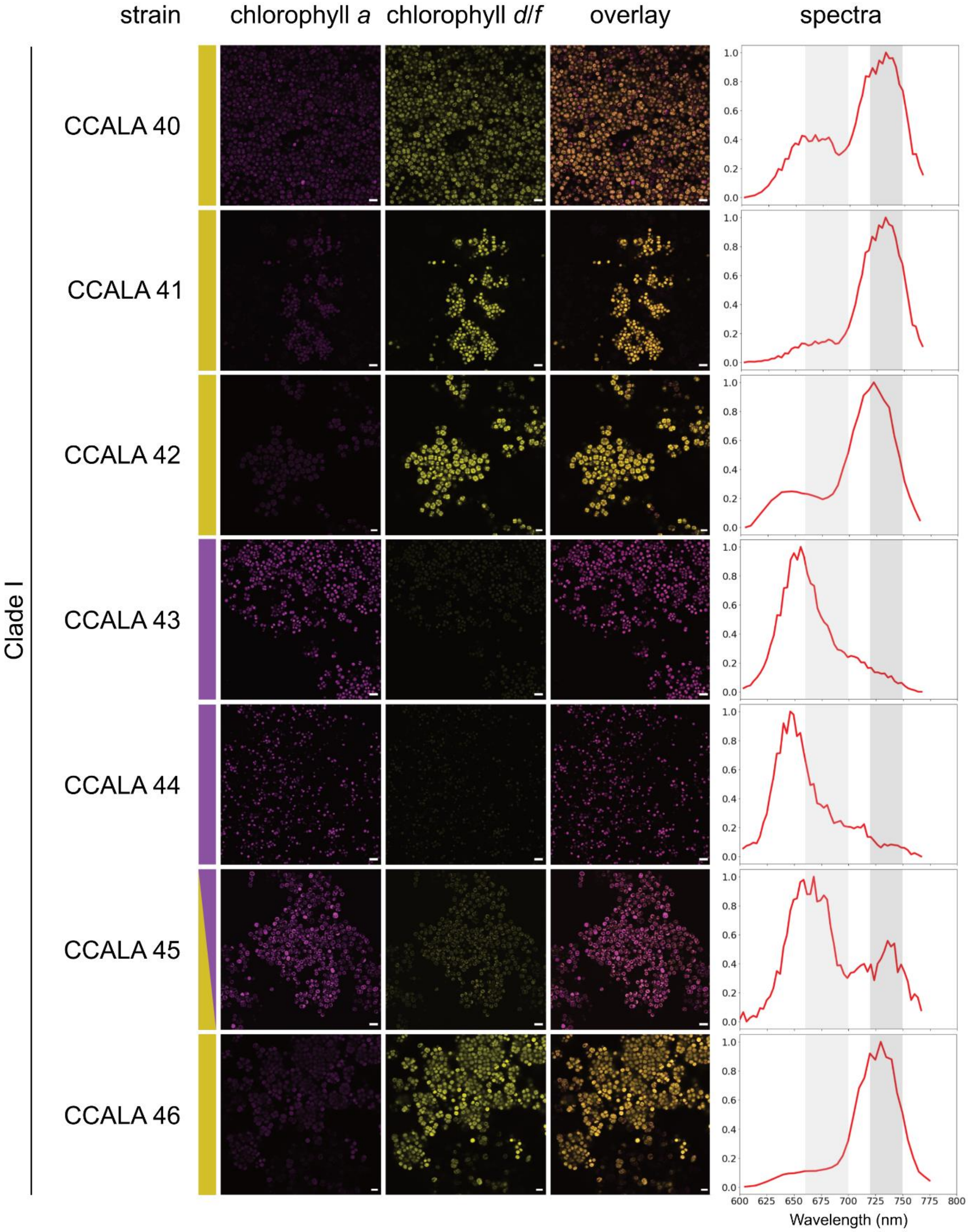

Figure S1. Capacity for FaRLiP in *Chroococcidiopsis*-like cyanobacteria *(figure continues below)*

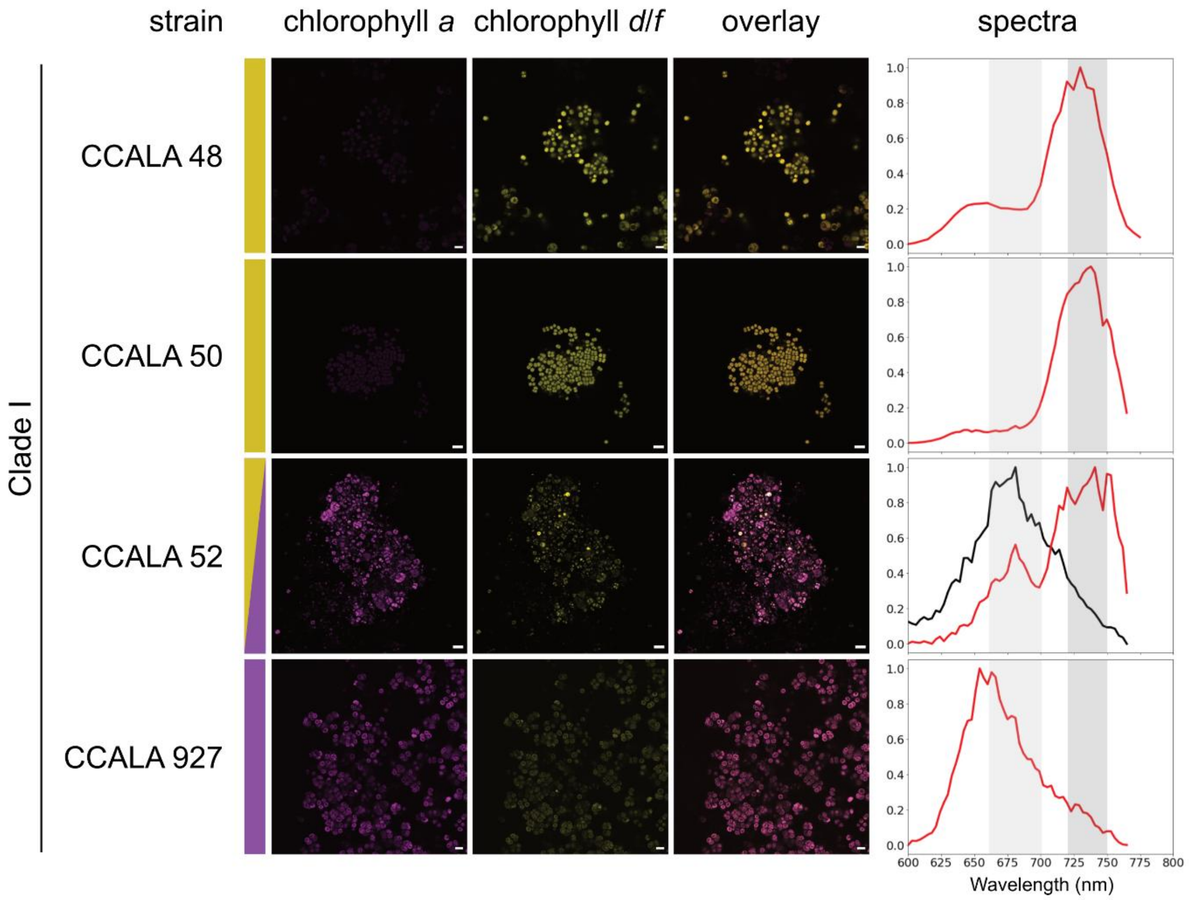

Figure S1. Capacity for FaRLiP in the *Chroococcidiopsidales*, as assessed by confocal microscopy. All strains contain chlorophyll *a* and phycobilisomes, as shown in magenta (fluorescence emission range 660–700 nm). Strains undergoing FaRLiP also show fluorescence around 720-780 nm (yellow), indicating the presence of chlorophylls *d*/*f*. FaRLiP-positive strains are marked with yellow bands; negative strains are marked with magenta bands. A minority of mixed or transitional results were also observed (mixed bands). The rightmost column shows a λ (wavelength)-scan for a minimum of 3 cells. Fluorescence emission channels are highlighted in grey bars.

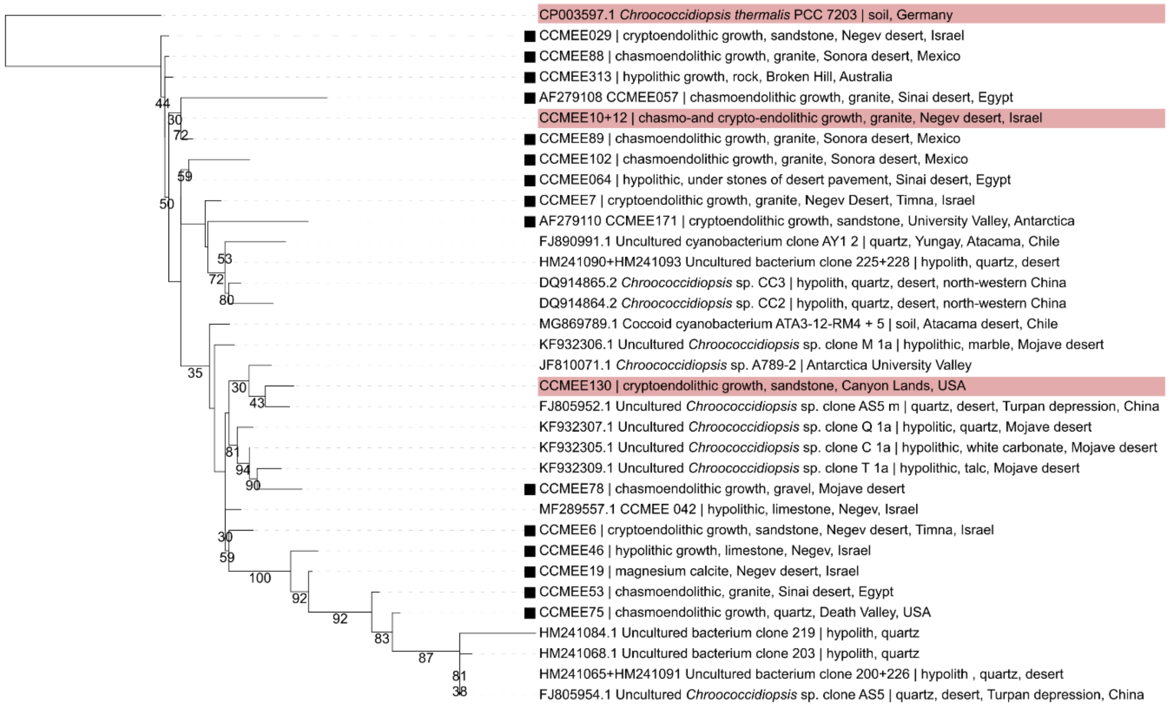

Figure S2. A 16S rRNA gene phylogeny of the Hot desert *Chroococcidiopsidales* (II), previously 'Hot desert *Chroococcidiopsis*. They are preferentially found as endoliths in arid, hot environments. Tree rooted with *Chroococcidiopsis thermalis* PCC 7203 as outgroup. Only three strains tested positive for FaRLiP (red highlight), while the majority of tested strains were negative (black squares). This is in contrast to the *Chroococcidiopsis sensu stricto* genus. Strains CCMEE 10 and CCMEE 12 were grouped together due to identical 16S rRNA gene sequences (I). Tree built with RaxML. Bootstrap values < 30 not shown.

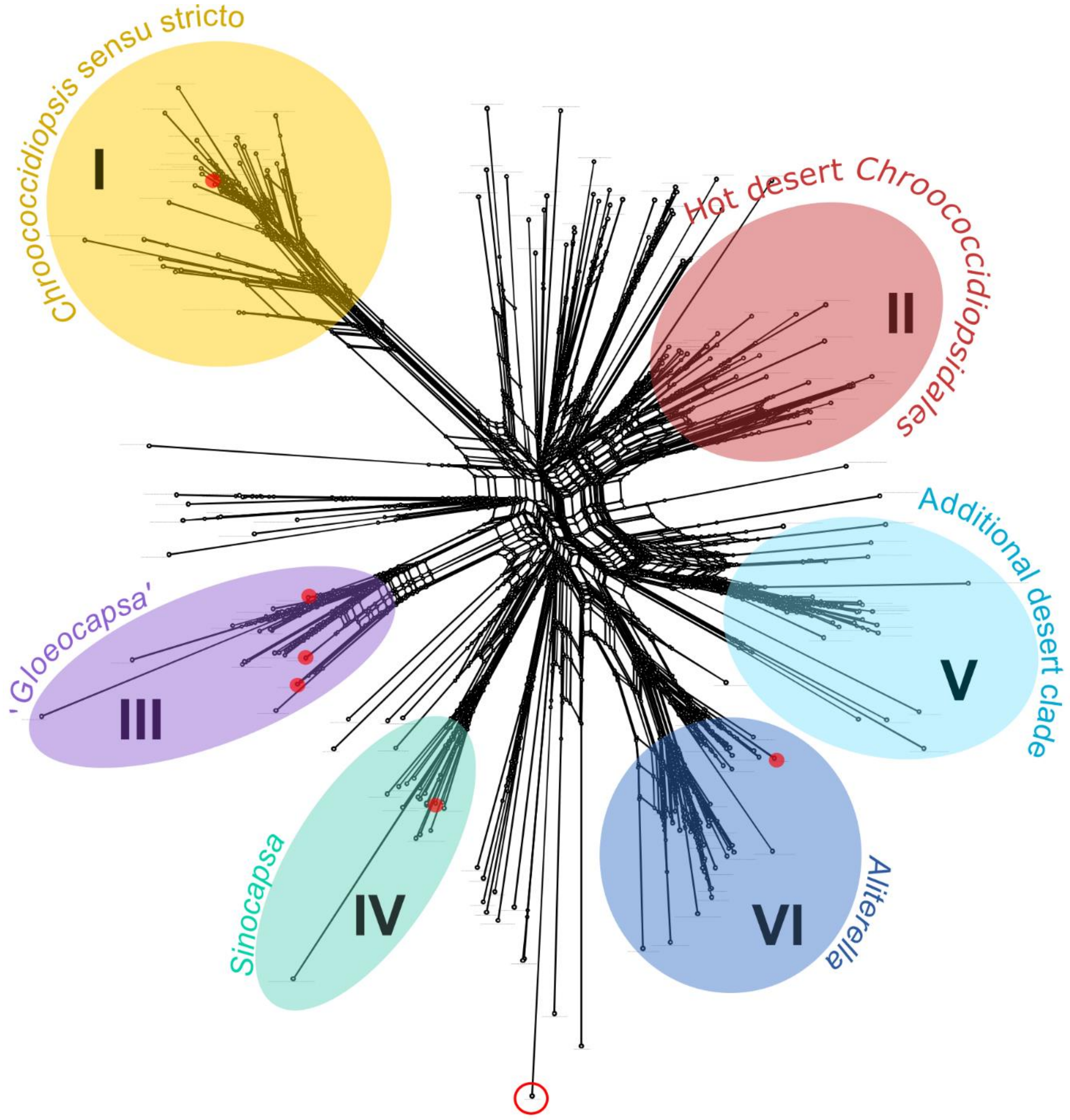

Figure S3. Network visualization of the main species clusters within the *Chroococcidiopsidales*. While not implying evolutionary relationships on its own, this sequence similarity-based network of the 16S rRNA gene alignment is fairly consistent with the corresponding phylogenetic tree (Figure 3). Same taxa were used. Filled open circles mark type strains, where available. The open red circle marks the distantly-related *Pleurocapsa* sp. PCC 7327. Minor clades *Haliplanktos* (VII) and *Pseudocyanosarcina* (VIII) are not shown. Default settings (NeighborNet, SplitsNetworkAlgorithm).

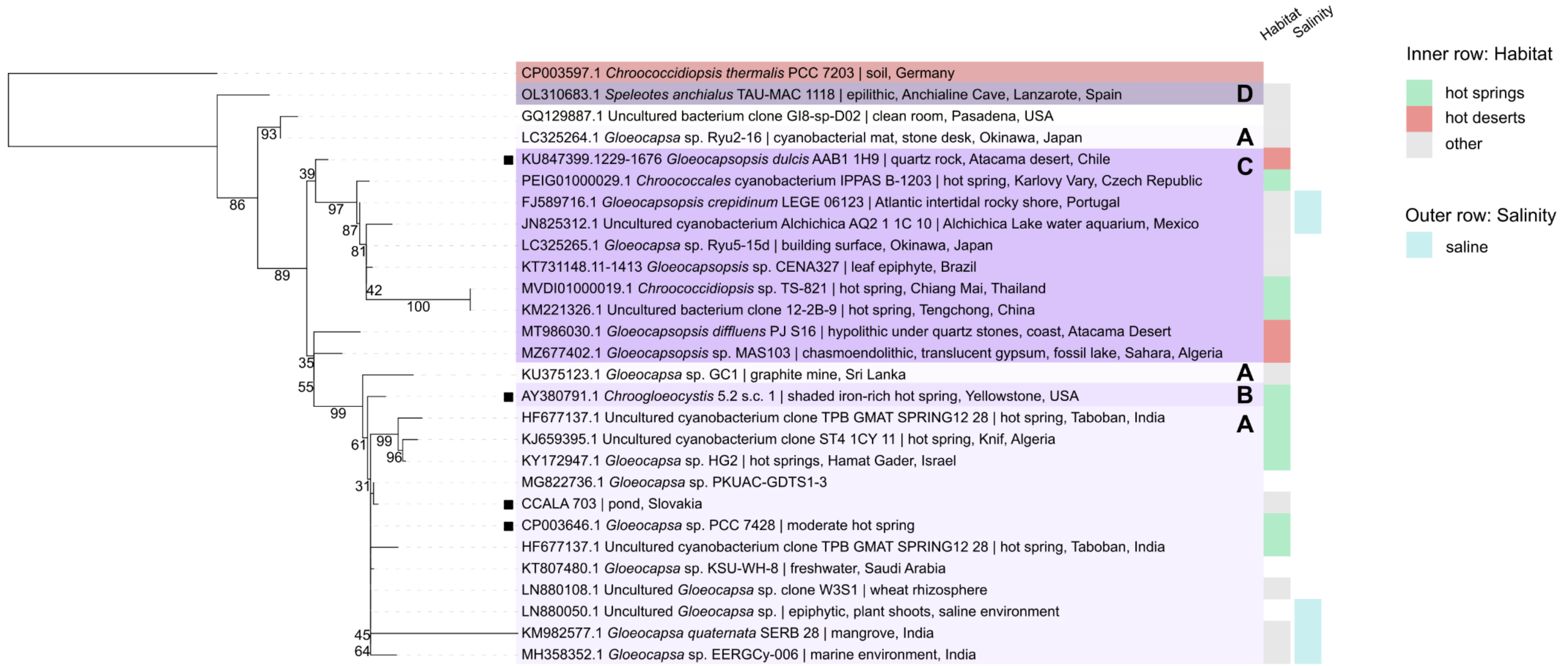

Figure S4. A 16S rRNA gene phylogeny of strains related to *Gloeocapsa* (III). They are preferentially found in hot springs or saline environments. The following genera have been defined: *Gloeocapsa* (A), *Chroogloeocystis* (B), *Gloeocapsopsis* (C), *Spelotes* (D). Tree rooted with *Chroococcidiopsis thermalis* PCC 7203 as outgroup. Black squares marks strains which tested negative for FaRLiP, either in the laboratory (CCALA 703) or by lacking the necessary genes in complete genomes. Tree built with RaxML. Bootstrap values < 30 not shown.

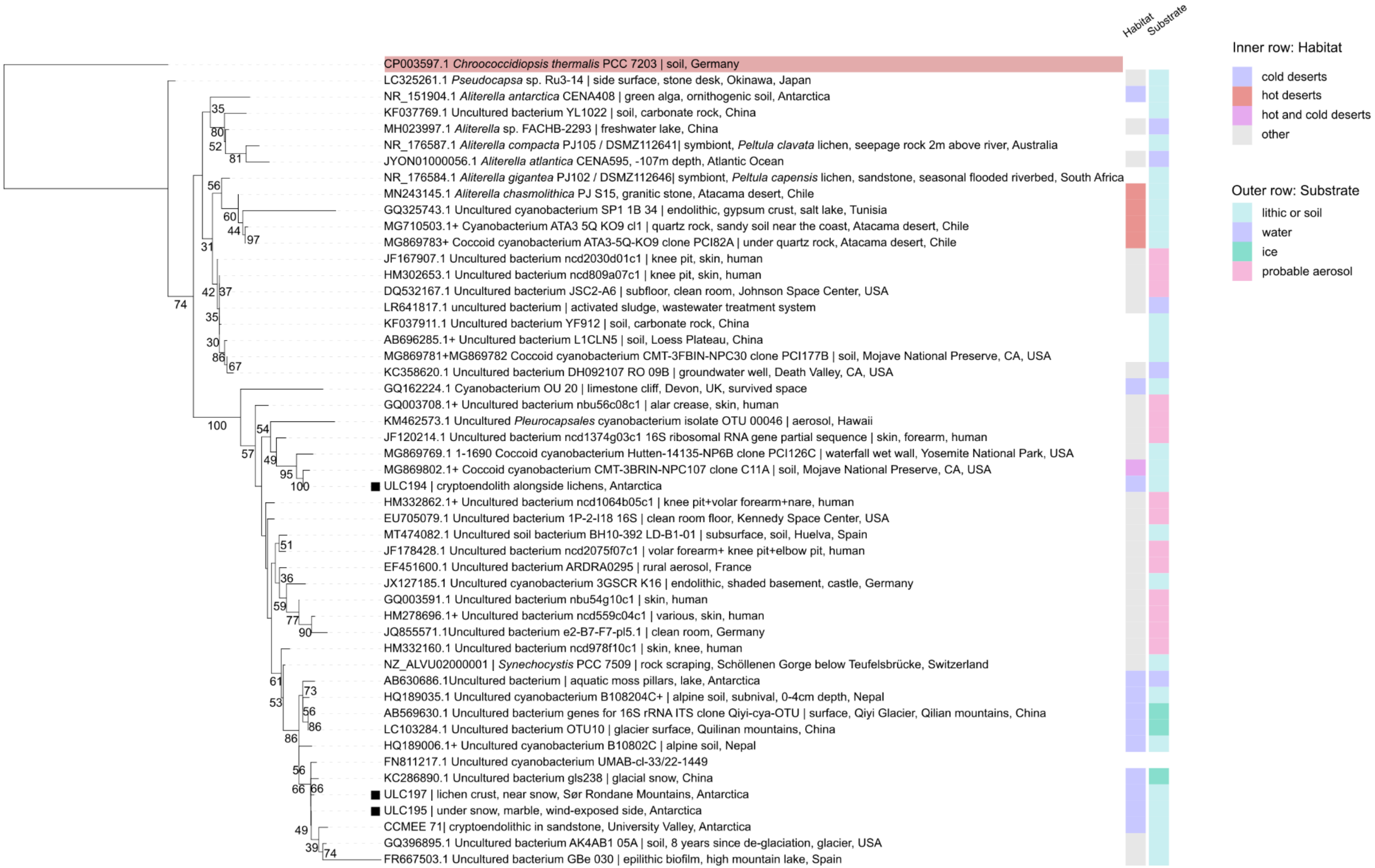

Figure S5. A 16S rRNA gene phylogeny of *Aliterella* (VI). These strains are often found in cold deserts, but are not limited to them. The tree was rooted with *Chroococcidiopsis thermalis* PCC 7203 as outgroup. Unlike the outgroup, none of the strains tested showed the capacity for FaRLiP (black squares). Built with RaxML. Bootstrap values <30 not shown.

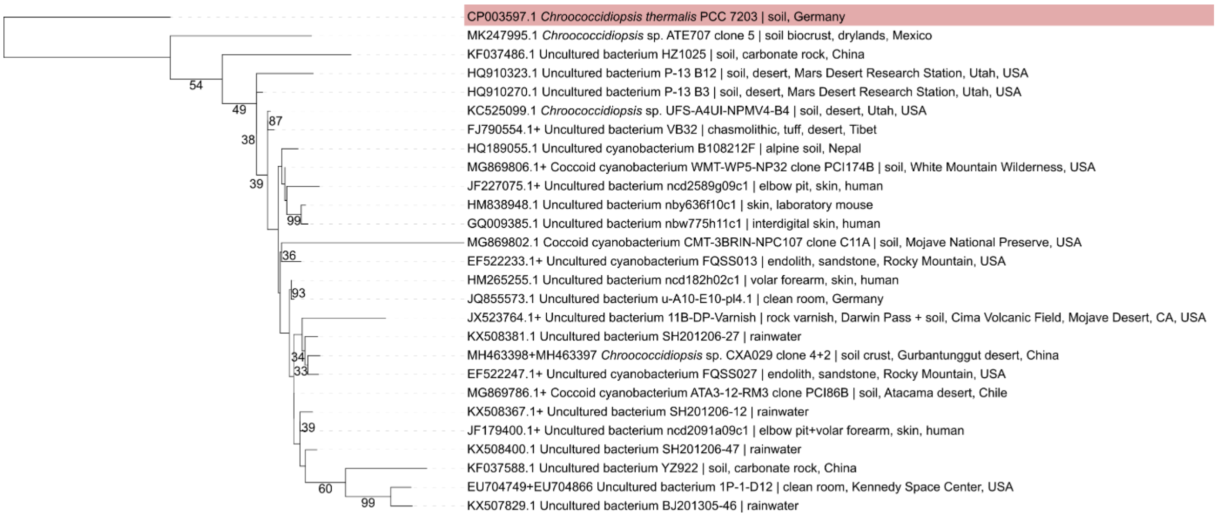

Figure S6. A 16S rRNA gene phylogeny of a yet-unnamed *Chroococcidiopsidales* taxon referred in this work as 'Additional desert clade' (V). No known strains from culture collections are present in this group, but 16S rRNA gene similarity hints at it being a distinct genus. The tree was rooted with *Chroococcidiopsis thermalis* PCC 7203 as outgroup. Built with RaxML. Bootstrap values <30 not shown.

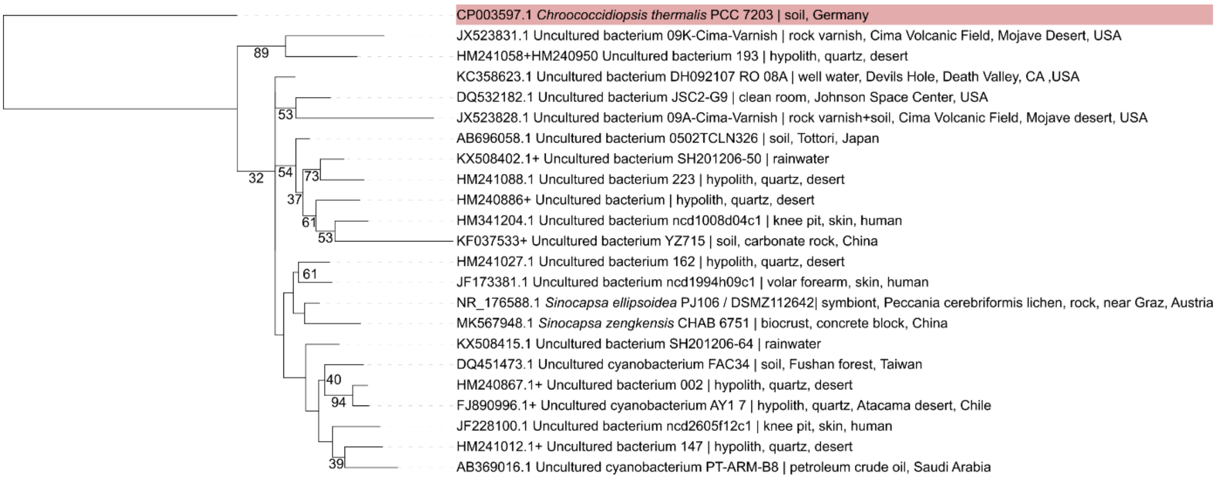

Figure S7. A 16S rRNA gene phylogeny of *Sinocapsa* (IV). There are currently only two cultured strain available (*Sinocapsa zengkensis* CHAB 6751 and *S. ellipsoidea* DSMZ112642). The tree was rooted with *Chroococcidiopsis thermalis* PCC 7203 as outgroup. Built with RaxML. Bootstrap values < 30 not shown.

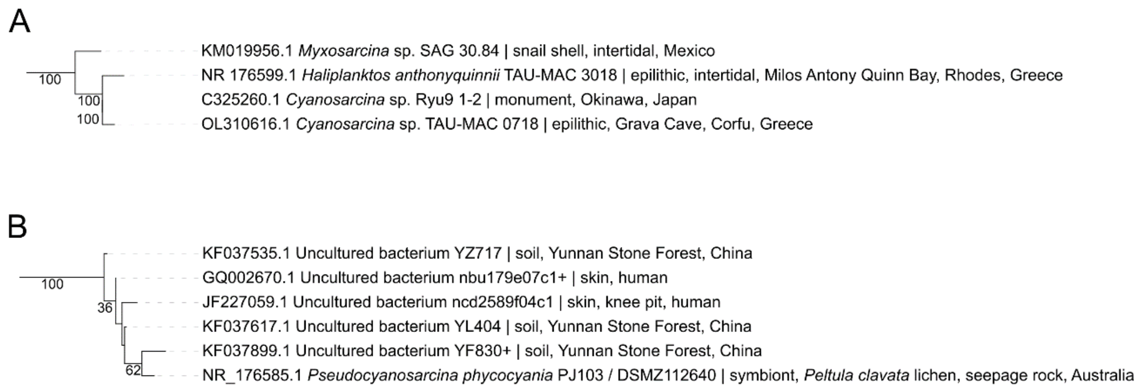

Figure S8. A 16S rRNA gene phylogeny of rare genera (A) *Haliplanktos* (VII) and (B) *Pseudocyanosarcina* (VIII). Insets from Figure 3a and 4. Built with RaxML. Bootstrap values < 30 not shown.

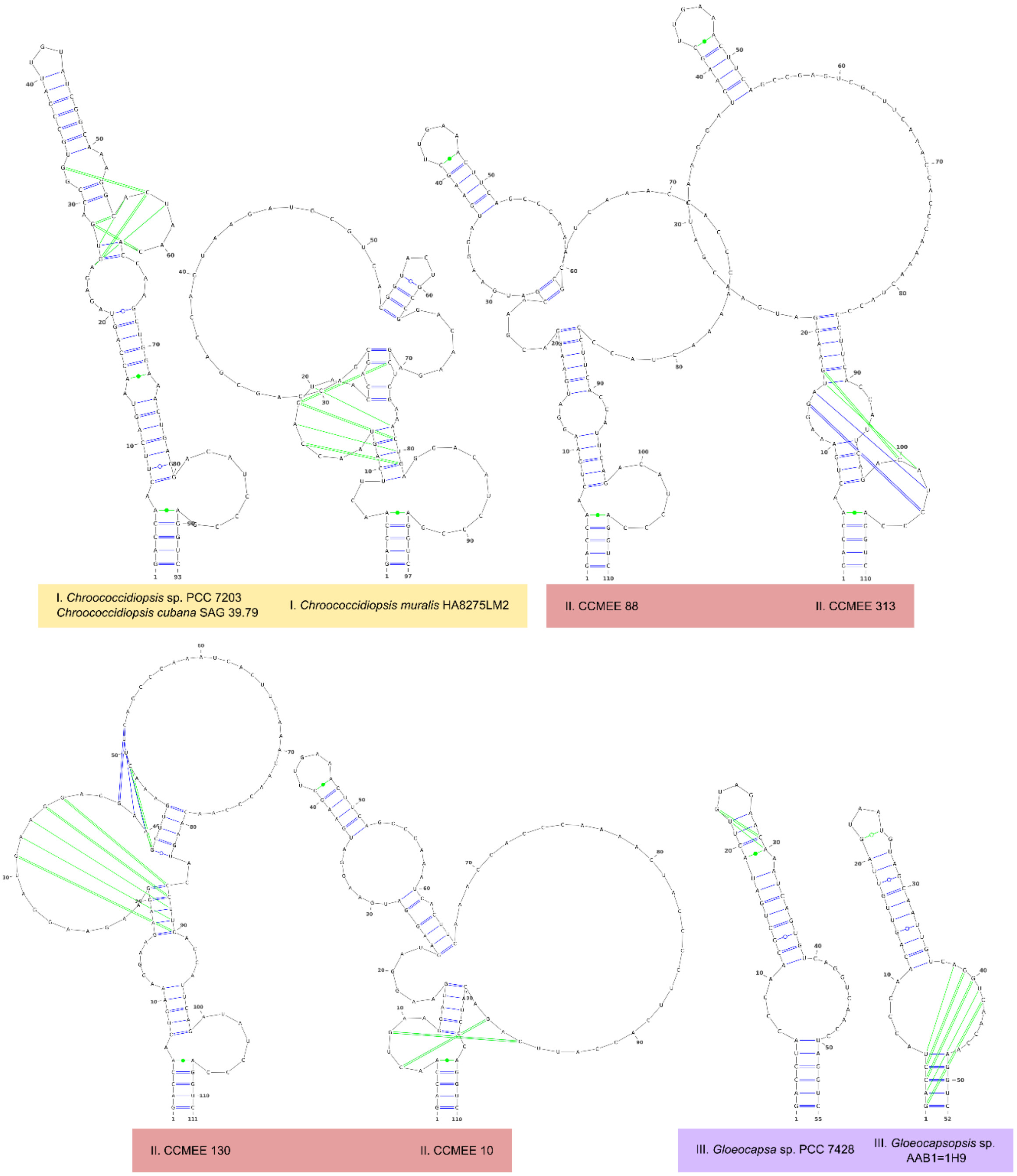

Figure S9. Predicted structures of the D1-D1’ loop in *Chroococcidiopsidales* lineages. *(Figure continues below)*

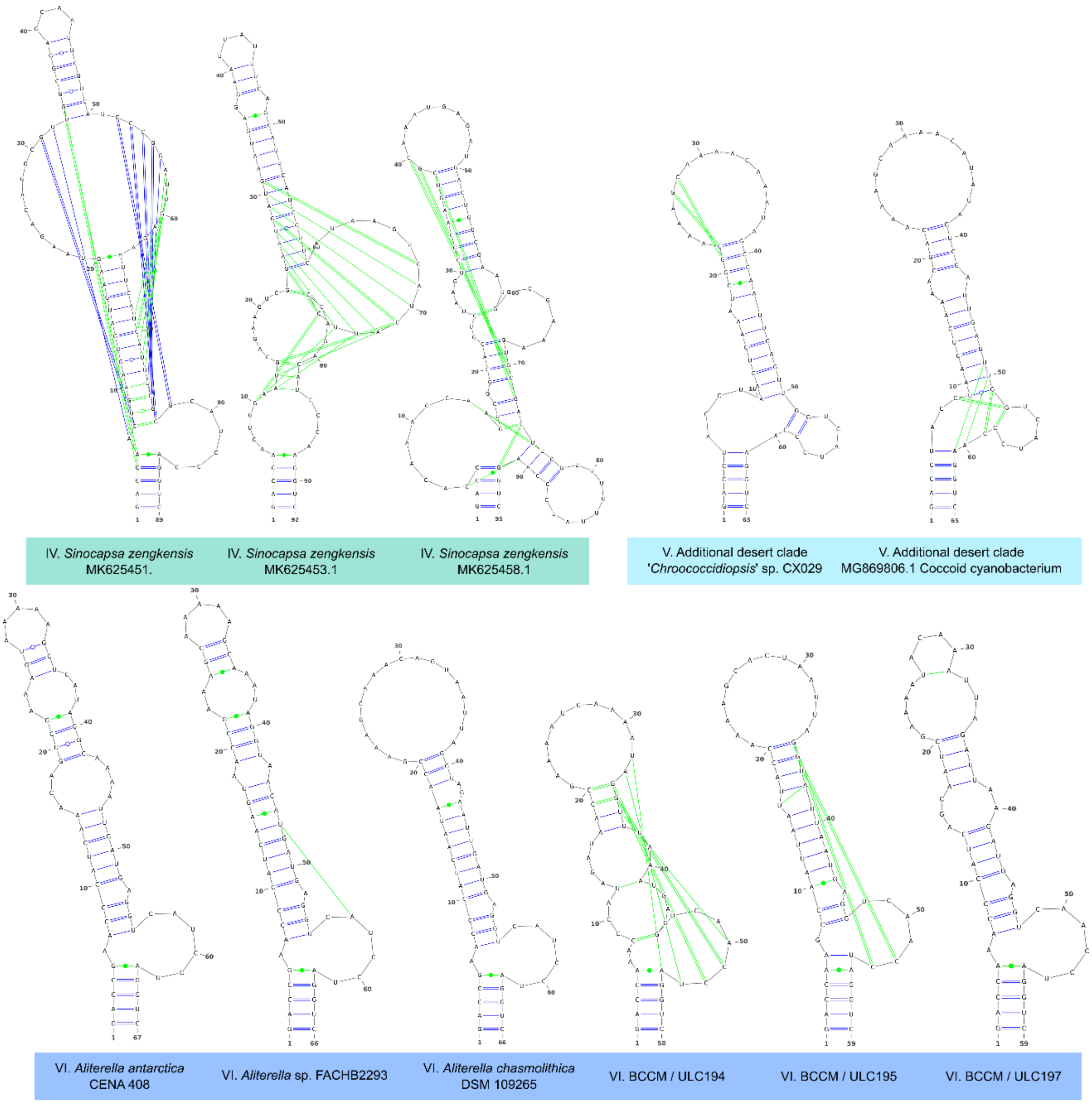

Figure S9. *(Figure continues above)* Predicted structures of the D1-D1' loop in *Chroococcidiopsidales* lineages. The structure of this ribosomal intergenic sequence (ITS) feature has been previously shown to correlate with cyanobacterial taxonomic units [17]. Green lines mark non-standard pairings and pseudoknot-type connections. Classification – I: *Chroococcidiopsis sensu stricto* (yellow); II: Hot desert *Chroococcidiopsidales* (red); III: '*Gloeocapsa*' (purple); IV: *Sinocapsa* (green); V: Additional desert clade (cyan); VI: *Aliterella* (blue).

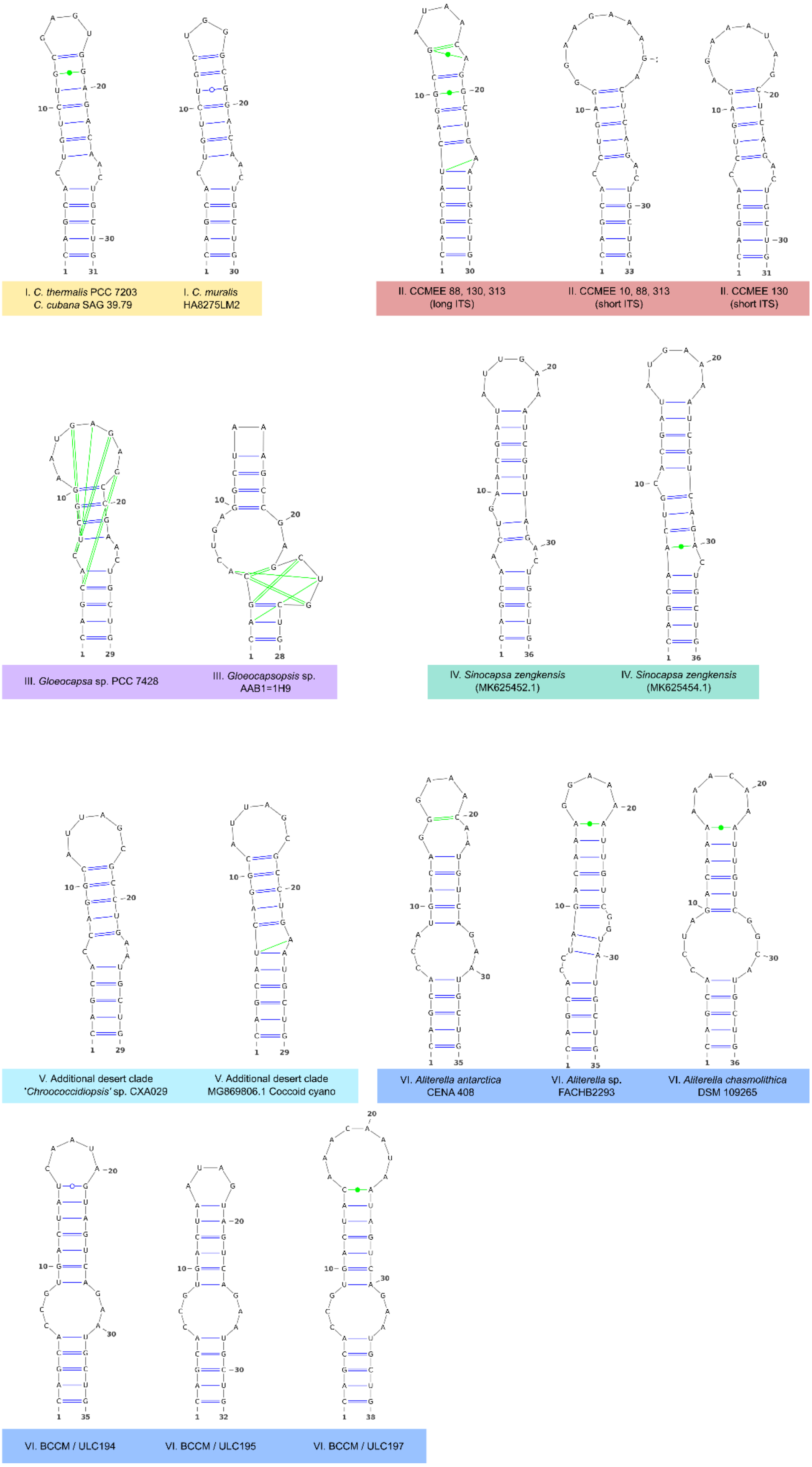

Figure S10. Predicted structures of the BoxB loop in *Chroococcidiopsidales* lineages. *(Legend continues below)*

Figure S10. Predicted structures of the BoxB loop in *Chroococcidiopsidales* lineages. The structure of this ribosomal intergenic sequence (ITS) feature has been previously shown to correlate with cyanobacterial taxonomic units [17]. Green lines mark non-standard pairings and pseudoknot-type connections. Classification – I: *Chroococcidiopsis sensu stricto* (yellow); II: Hot desert *Chroococcidiopsidales* (red); III: *'Gloeocapsa'* (purple); IV: *Sinocapsa* (green); V: Additional desert clade (cyan); VI: *Aliterella* (blue).

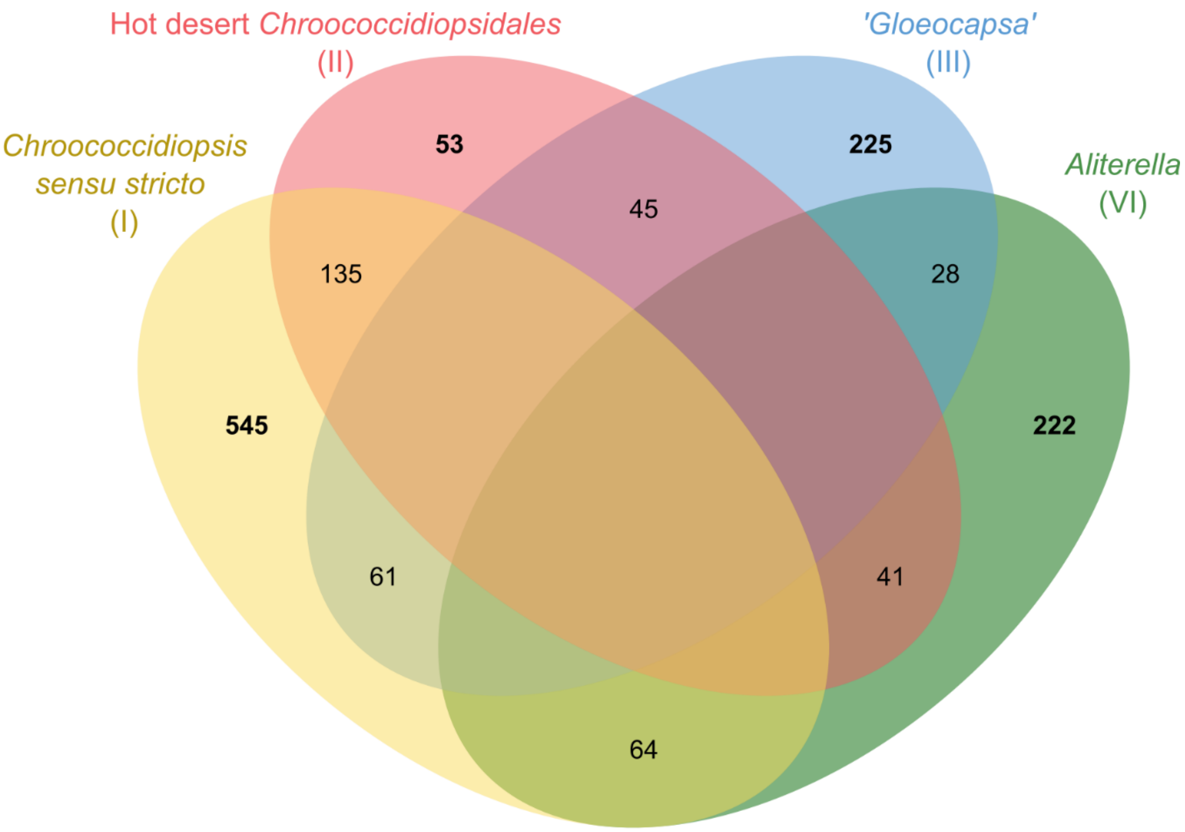

Figure S11. Venn diagram illustrating shared groups of orthologous genes (orthogroups) within the *Chroococcidiopsidales*. There are more clade-specific orthogroups (in bold) than orthogroups shared between any two clades. Only one incomplete MAG (ignimbrite12) was available for clade II (Hot desert *Chroococcidiopsidales*). This likely explains the fewer clade-specific genes recovered. Created with OrthoVenn2.

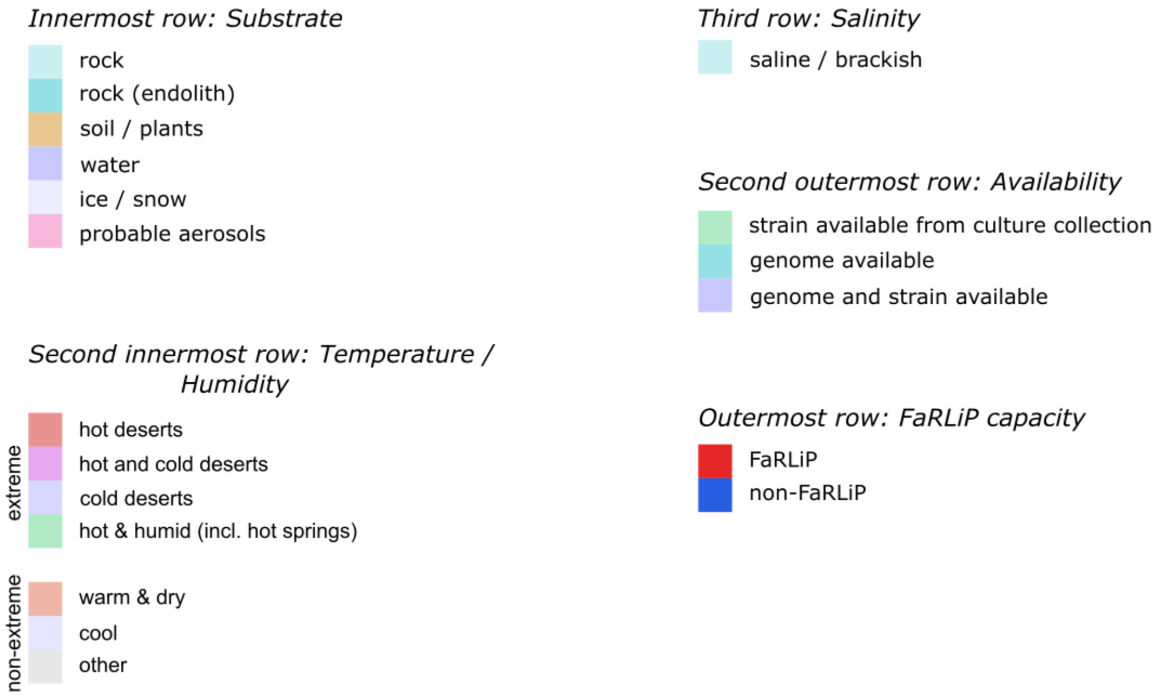

Guidelines:

For visualizing a sequence label:
Hover cursor over branch of interest or associated color strip.
For visualizing all sequence labels:
Control panel > Basic > Labels > Display
For visualizing bootstrap values:
Control Panel > Advanced > Branch metadata display > Bootstraps / metadata: > Display > Text

Figure S12. Legend and brief guidelines for the expanded online version of Figure 4, which can be accessed at https://itol.embl.de/tree/13013366682000716621291 17.

Supplementary Text 1

***'Gloeocapsa'*: hot springs/salinity specialist (Lineage III)**

Although closely related to *Chroococcidiopsis sensu stricto* (I), strains in this clade are rarely mislabeled as such. This is probably due to their very distinct morphologies (layered extracellular polysaccharide sheath, with some EPS reaching as far as 1 µm) [18–21]. This has been previously referred to as 'F layer' [22]. Lineage III includes genera *Gloeocapsopsis*, *Chroogloeocystis*, *Gloeocapsa* and *Speleotes* (Figure S4) of varied taxonomic validity [19, 20, 23–25].

Strains are often found in hot springs or saline environments (Figure S4). The majority have been isolated from aqueous environments, and even strains isolated from the Atacama Desert have been sampled from close to the coast. This suggests that adaptations against desiccation in *'Gloeocapsa'* (III) might be relatively short term. However, one of these strains, AAB1 (equivalent to 1H9 and UTEX B3054) has nevertheless survived desiccation for two years [18].

The need to survive desiccation and UV stress is also clear at the genome level (Table S9). There are clade-specific genes involved in the biosynthesis and recycling of trehalose and cellulose, as previously published [18, 26]; also, there are 1-2 clade-specific genes associated with carotenoid biosynthesis, including a putative canthaxanthin synthase. This ketocarotenoid was found to be enriched in under high light in *'Chroococcidiopsis'* sp. TS-821 (belonging to lineage III) [27]. Biotic factors may also provide selective pressure on the genome. There are three additional genes encoding an integral membrane protein putatively assigned as an aminoethylphosphonate transporter (Table S9). Aminoethylphosphonate is an organic phosphorus source with a difficult to cleave C-P bond, which is a viable phosphorus source for the few cyanobacteria which can use it in marine and hot spring biofilm environments [28, 29]. This may reflect the typical biofilm habitat of '*Gloeocapsa'* strains, alongside the extra genes for the metabolism of nucleotides, amino acids, and compounds with a cyano (C-N) bond.

Supplementary Text 2

***Aliterella*: cold/hot and cold desert/'aerosolization' specialists (Lineage VI)**

This genus has been previously defined as 'Cold desert *Chroococcidiopsis*', and indeed, many associated sequences have been recovered from cold environments, such as mountain glaciers and Antarctica, occasionally with cells found directly on the snow [9, 10]. However, the full range of environments includes hot deserts like the Atacama, non-extreme habitats including soil, and hostile anthropogenic environments such as clean rooms for spacecraft assembly (Figure S5).

Chroococcidiopsis has been defined as a common clean room contaminant [30, 31], but this study shows that many clean room sequences actually fall under the *Aliterella* (VI) classification [32] or the related 'Additional desert clade' (V). It is very likely that adaptations to survive low water pressure in cold environments may pre-adapt these strains to clean room survival and, more generally, to aerosolization.

A morphological characteristic of the *Aliterella* genus appears to be its ability to produce thick, clear mucilage which encases the cells, and may be solid enough to maintain its shape if cells are mechanically removed [32–34]. It has been hypothesized that it protects the cells from UV radiation, temperature extremes and even vacuum, in the case of a strain which survived space [34, 35]. It is probably this protective layer which has led to some *Aliterella* strains being putatively identified as similar to '*Gloeocapsa'* (III) [34]. As such, it can be suspected that some early reports of *Gloeocapsa* in Antarctic rocks could be assigned to *Aliterella* [36].

Cold tolerance is very likely a characteristic of the *Aliterella* genus. Fittingly, many of the clade-specific genes identified are associated with lipid synthesis, in particular the synthesis of unsaturated fatty acids and of glycerophospholipids (Table S9) [37]. For some of these genes, very few cyanobacterial homologues exist, and there is high similarity to sequences in the distantly related *Verrucomicrobia*. This group has also been found in cold environments [38]. It is possible that this highlights a niche shift following horizontal gene transfer event(s). Moreover, *Aliterella* has a gene set for the dissimilatory nitrate reduction pathway, putatively associated with energy conservation and temporary survival in low-oxygen/anoxic zones [39, 40] and rarely found in cyanobacteria.

Supplementary text 3

Hot desert *Chroococcidiopsidales*: hot desert specialists (Lineage II)

Strains and sequences from this group have commonly been sampled from within hot (as opposed to arctic/alpine) deserts. These include the Atacama, Negev and Sinai deserts. Even in Yungay, the hyperarid core of the Atacama Desert, one of the driest places on Earth, clade II sequences were recovered from cyanobacteria-dominated patches under (sequence FJ890991) [41], or inside rocks (MAG ignimbrite12) [42]. It is likely that many previous reports of *Chroococcidiopsis* in hyperarid deserts may refer to this clade, but sequences from other lineages, such as *Sinocapsa* (IV) and *Aliterella* (VI), also appear common (Figure 4).

There has been considerable laboratory work on this clade, typically under the name '*Chroococcidiopsis*' or 'Hot desert *Chroococcidiopsis*'. Strains have survived multi-year desiccation assays (although at higher water pressures than was tested for *Gloeocapsa*) (Supplementary Text 1) [43], as well as UV-C and high ionizing radiation [44], including when being exposed to space or simulated Martian conditions [45]. The biofilm growth form of these cyanobacteria appears to make them especially resistant to high-energy radiation. Cells are embedded within a lipid-rich EPS matrix, which likely enhances survival [45]. Some of the clade-specific glycotransferase sequences found in this study were assigned to 'lipopolysacharide biosynthesis' and might be involved in EPS production. In addition, sequences labeled as 'endonuclease' and 'NAD(P)H-dependent oxidoreductase' could potentially be associated with limiting oxidative stress (Tables S9, S10). Future comparative studies could also consider the genome *Chroococcidiopsis* sp. CCMEE 29, which has recently become available (NCBI code ASM2355837v1).

Supplementary Text 4

Chroococcidiopsis sensu stricto: generalists (Lineage I)

The environments from which members of the genus have been recovered are highly diverse. This is consistent with them being generalists, as opposed to specialists. At the cellular level, a ribbon-like structure of the cell wall (‘S-layer’) appears to be unique to this genus, though data are limited [46].

One main theme that emerges from looking at clade-specific genes, through genome comparisons, is a putative connection to microoxic environments. The *Chroococcidiopsis sensu stricto* (I) genomes studied include *hox* genes (encoding the components for a bidirectional hydrogenase), *nifJ* (metabolically associated with the hydrogenase, used in fermentation) and two likely *sox* genes (sulfite oxidases, typically expressed in microoxic environments), as well as a gene putatively involved in the synthesis of molybdopterin (a cofactor necessary for sulfate oxidases as well as for xanthine dehydrogenase, one of the specific purine genes) (Table S9). A hydrogenase has been shown to take up hydrogen when cells are exposed to light in *C. thermalis* CALU 758 [47]. It does not appear to be present in extremophilic *Chroococcidiopsidales* lineages [48]. Considering that *Chroococcidiopsis sensu stricto* is the one *Chroococcidiopsidales* clade which has near universally maintained the FaRLiP cluster, it is possible that far-red photosynthesis and microoxic adaptations complement each other, possibly providing a selective advantage in microenvironments such as soil and the inside of microbial mats.

Supplementary references

1. Nürnberg DJ, Morton J, Santabarbara S, Telfer A, Joliot P, Antonaru LA, et al. Photochemistry beyond the red limit in chlorophyll f–containing photosystems. *Science* 2018; **360**: 1210–1213.
2. Antonaru LA, Cardona T, Larkum AWD, Nürnberg DJ. Global distribution of a chlorophyll f cyanobacterial marker. *ISME J* 2020; **14**: 2275–2287.
3. Nübel U, Garcia-Pichel F, Muyzer G. PCR primers to amplify 16S rRNA genes from cyanobacteria. *Appl Environ Microbiol* 1997; **63**: 3327–3332.
4. Ferris MJ, Muyzer G, Ward DM. Denaturing gradient gel electrophoresis profiles of 16S rRNA-defined populations inhabiting a hot spring microbial mat community. *Appl Environ Microbiol* 1996; **62**: 340–346.
5. Janse I, Meima M, Kardinaal WEA, Zwart G. High-resolution differentiation of Cyanobacteria by using rRNA-Internal Transcribed Spacer Denaturing Gradient Gel Electrophoresis. *Appl Environ Microbiol* 2003; **69**: 6634–6643.
6. Neilan BA, Stuart JL, Goodman AE, Cox PT, Hawkins PR. Specific amplification and restriction polymorphisms of the cyanobacterial rRNA operon spacer region. *Syst Appl Microbiol* 1997; **20**: 612–621.
7. Yarza P, Yilmaz P, Pruesse E, Glöckner FO, Ludwig W, Schleifer KH, et al. Uniting the classification of cultured and uncultured bacteria and archaea using 16S rRNA gene sequences. *Nat Rev Microbiol* 2014; **12**: 635–645.
8. Jung P, Brust K, Schultz M, Büdel B, Donner A, Lakatos M. Opening the gap: rare lichens with rare cyanobionts – unexpected cyanobiont diversity in cyanobacterial lichens of the order Lichinales. *Front Microbiol* 2021; **12**: 1–24.
9. Fagliarone C, Mosca C, Ubaldi I, Verseux C, Baqué M, Wilmotte A, et al. Avoidance of protein oxidation correlates with the desiccation and radiation resistance of hot and cold desert strains of the cyanobacterium Chroococcidiopsis. *Extremophiles* 2017; **21**: 981–991.
10. Bahl J, Lau MCY, Smith GJD, Vijaykrishna D, Cary SC, Lacap DC, et al. Ancient origins determine global biogeography of hot and cold desert cyanobacteria. *Nat Commun* 2011; **2**: 1–6.
11. Pointing SB, Warren-Rhodes KA, Lacap DC, Rhodes KL, McKay CP. Hypolithic community shifts occur as a result of liquid water availability along environmental gradients in China's hot and cold hyperarid deserts. *Environ Microbiol* 2007; **9**: 414–424.
12. Fewer D, Friedl T, Büdel B. Chroococcidiopsis and heterocyst-differentiating cyanobacteria are each other's closest living relatives. *Mol Phylogenet Evol* 2002; **23**: 82–90.
13. Zhang ZC, Li ZK, Yin YC, Li Y, Jia Y, Chen M, et al. Widespread occurrence and unexpected diversity of red-shifted chlorophyll producing cyanobacteria in humid subtropical forest ecosystems. *Environ Microbiol* 2019; **21**: 1497–1510.
14. Allen MA, Goh F, Burns BP, Neilan BA. Bacterial, archaeal and eukaryotic diversity of smooth and pustular microbial mat communities in the hypersaline lagoon of Shark Bay. *Geobiology* 2009; **7**: 82–96.
15. Schipper K, Al Muraikhi M, Alghasal GSHS, Saadaoui I, Bounnit T, Rasheed R, et al. Potential of novel desert microalgae and cyanobacteria for commercial applications and CO2 sequestration. *J Appl Phycol* 2019; **31**: 2231–2243.
16. Cumbers J, Rothschild LJ. Salt tolerance and polyphyly in the cyanobacterium Chroococcidiopsis (Pleurocapsales). *J Phycol* 2014; **50**: 472–482.

17. Iteman I, Rippka R, De Marsac NT, Herdman M. Comparison of conserved structural and regulatory domains within divergent 16S rRNA-23S rRNA spacer sequences of cyanobacteria. *Microbiology* 2000; **146**: 1275–1286.

18. Azua-Bustos A, Zúñiga J, Arenas-Fajardo C, Orellana M, Salas L, Rafael V. Gloeocapsopsis AAB1, an extremely desiccation-tolerant cyanobacterium isolated from the Atacama Desert. *Extremophiles* 2014; **18**: 61–74.

19. Vitor R, Rui S, Ângela B, Arlete S, Santos CL, Miguel L, et al. Characterization of an intertidal cyanobacterium that constitutes a separate clade together with thermophilic strains. *Eur J Phycol* 2010; **45**: 394–403.

20. Brown II, Mummey D, Cooksey KE. A novel cyanobacterium exhibiting an elevated tolerance for iron. *FEMS Microbiol Ecol* 2005; **52**: 307–314.

21. Sun CN. Electron Microscope observations on Gloeocapsa sp. *Bull Torrey Bot Club* 1961; **88**: 106–110.

22. Hayashi NR, Yuwadee P, Hirofumi N, Masaharu I, Yasuo I, Tohru K. Isolation and cultivation of thermophilic cyanobacteria from hot springs of Northern Thailand. *J Ferment Bioeng* 1994; **78**: 179–181.

23. Komárek J, Kaštovský J, Mareš J, Johansen JR. Taxonomic classification of cyanoprokaryotes (cyanobacterial genera) 2014, using a polyphasic approach. *Preslia* 2014; **86**: 295–335.

24. Panou M, Gkelis S. Unravelling unknown cyanobacteria diversity linked with HCN production. *Mol Phylogenet Evol* 2022; **166**: 107322.

25. Strunecký O, Ivanova AP, Mareš J. An updated classification of cyanobacterial orders and families based on phylogenomic and polyphasic analysis. *J Phycol* 2022; **51**: 12–51.

26. Urrejola C, Alcorta J, Salas L, Vásquez M, Polz MF, Vicuña R, et al. Genomic features for desiccation tolerance and sugar biosynthesis in the extremophile Gloeocapsopsis sp. UTEX B3054. *Front Microbiol* 2019; **10**: 1–11.

27. Li M, Calteau A, Semchonok DA, Witt TA, Nguyen JT, Sassoon N, et al. Physiological and evolutionary implications of tetrameric photosystem I in cyanobacteria. *Nat Plants* 2019; **5**: 1309–1319.

28. Gomez-Garcia MR, Davison M, Blain-Hartnung M, Grossman AR, Bhaya D. Alternative pathways for phosphonate metabolism in thermophilic cyanobacteria from microbial mats. *ISME J* 2011; **5**: 141–149.

29. Quinn JP, Kulakova AN, Cooley NA, McGrath JW. New ways to break an old bond: The bacterial carbon-phosphorus hydrolases and their role in biogeochemical phosphorus cycling. *Environ Microbiol* 2007; **9**: 2392–2400.

30. Moissl-Eichinger C, Auerbach AK, Probst AJ, Mahnert A, Tom L, Piceno Y, et al. Quo vadis? Microbial profiling revealed strong effects of cleanroom maintenance and routes of contamination in indoor environments. *Sci Rep* 2015; **5**: 1–13.

31. Mahnert A, Vaishampayan P, Probst AJ, Auerbach A, Moissl-Eichinger C, Venkateswaran K, et al. Cleanroom maintenance significantly reduces abundance but not diversity of indoor microbiomes. *PLoS One* 2015; **10**: 1–20.

32. Rigonato J, Gama WA, Alvarenga DO, Branco LHZ, Brandini FP, Genuário DB, et al. Aliterella atlantica gen. nov., sp. nov., and Aliterella antarctica sp. nov., novel members of coccoid Cyanobacteria. *Int J Syst Evol Microbiol* 2016; **66**: 2853–2861.

33. Zhang Q, Zheng L, Li T, Li R, Song L. Aliterella shaanxiensis (Aliterellaceae), a new coccoid cyanobacterial species from China. *Phytotaxa* 2018; **374**: 211–220.

34. Olsson-Francis K, De La Torre R, Cockell CS. Isolation of novel extreme-tolerant cyanobacteria from a rock-dwelling microbial community by using exposure to Low Earth Orbit. *Appl Environ Microbiol* 2010; **76**: 2115–2121.

35. Cockell CS, Rettberg P, Rabbow E, Olsson-Francis K. Exposure of phototrophs to 548 days in low Earth orbit: Microbial selection pressures in outer space and on early earth. *ISME J* 2011; **5**: 1671–1682.

36. Friedmann EI, Ocampo R. Endolithic blue-green algae in the Dry Valleys : primary producers in the Antarctic Desert ecosystem. *Science* 1976; **193**: 1247–1249.

37. Srinivas TNR, Nageswara Rao SSS, Vishnu Vardhan Reddy P, Pratibha MS, Sailaja B, Kavya B, et al. Bacterial diversity and bioprospecting for cold-active lipases, amylases and proteases, from culturable bacteria of Kongsfjorden and Ny-Ålesund, Svalbard, Arctic. *Curr Microbiol* 2009; **59**: 537–547.

38. Waschulin V, Borsetto C, James R, Newsham KK, Donadio S, Corre C, et al. Biosynthetic potential of uncultured Antarctic soil bacteria revealed through long-read metagenomic sequencing. *ISME J* 2022; **16**: 101–111.

39. Marchant HK, Lavik G, Holtappels M, Kuypers MMM. The fate of nitrate in intertidal permeable sediments. *PLoS One* 2014; **9**: 1–12.

40. Kamp A, De Beer D, Nitsch JL, Lavik G, Stief P. Diatoms respire nitrate to survive dark and anoxic conditions. *Proc Natl Acad Sci U S A* 2011; **108**: 5649–5654.

41. Lacap DC, Warren-Rhodes KA, McKay CP, Pointing SB. Cyanobacteria and chloroflexi-dominated hypolithic colonization of quartz at the hyper-arid core of the Atacama Desert, Chile. *Extremophiles* 2011; **15**: 31–38.

42. Crits-Christoph A, Robinson CK, Ma B, Ravel J, Wierzchos J, Ascaso C, et al. Phylogenetic and functional substrate specificity for endolithic microbial communities in hyper-arid environments. *Front Microbiol* 2016; **7**: 1–15.

43. Billi D. Subcellular integrities in Chroococcidiopsis sp. CCMEE 029 survivors after prolonged desiccation revealed by molecular probes and genome stability assays. *Extremophiles* 2009; **13**: 49–57.

44. Verseux C, Baqué M, Cifariello R, Fagliarone C, Raguse M, Moeller R, et al. Evaluation of the resistance of Chroococcidiopsis spp. to sparsely and densely ionizing irradiation. *Astrobiology* 2017; **17**: 118–125.

45. Billi D, Staibano C, Verseux C, Fagliarone C, Mosca C, Baqué M, et al. Dried biofilms of desert strains of Chroococcidiopsis survived prolonged exposure to space and Mars-like conditions in Low Earth Orbit. *Astrobiology* 2019; **19**: 1008–1017.

46. Büdel B, Rhiel E. A new cell wall structure in a symbiotic and a free-living strain of the blue-green alga genus Chroococcidiopsis (Pleurocapsales). *Arch Microbiol* 1985; **143**: 117–121.

47. Serebryakova LT, Sheremetieva ME, Lindblad P. H2-uptake and evolution in the unicellular cyanobacterium Chroococcidiopsis thermalis CALU 758. *Plant Physiol Biochem* 2000; **38**: 525–530.

48. Kothari A, Potrafka R, Garcia-Pichel F. Diversity in hydrogen evolution from bidirectional hydrogenases in cyanobacteria from terrestrial, freshwater and marine intertidal environments. *J Biotechnol* 2012; **162**: 105–114.
